# Skills to Enhance Positivity in adolescents at risk for suicide: Protocol for a randomized controlled trial

**DOI:** 10.1371/journal.pone.0287285

**Published:** 2023-10-20

**Authors:** Shirley Yen, Nazaret Suazo, Jackson Doerr, Natalia Macrynikola, Leanna S. Villarreal, Sophia Sodano, Kimberly H. M. O’Brien, Jennifer C. Wolff, Christopher Breault, Brandon E. Gibb, Rani Elwy, Christopher W. Kahler, Megan Ranney, Richard Jones, Anthony Spirito

**Affiliations:** 1 Department of Psychiatry, Beth Israel Deaconess Medical Center, Boston, MA, United States of America; 2 Massachusetts Mental Health Center, Boston, MA, United States of America; 3 Department of Psychiatry, Harvard Medical School, Boston, MA, United States of America; 4 Department of Psychiatry and Human Behavior, Alpert Medical School of Brown University, Providence, RI, United States of America; 5 Northeastern University, Boston, MA, United States of America; 6 Bradley Hospital, Providence, RI, United States of America; 7 Boston Children’s Hospital, Boston, MA, United States of America; 8 Rhode Island Hospital, Providence, RI, United States of America; 9 Center for Behavioral and Preventive Medicine, The Miriam Hospital, Providence, RI, United States of America; 10 Department of Psychology, Binghamton University, Binghamton, NY, United States of America; 11 Department of Behavioral and Social Sciences, Brown University School of Public Health, Providence, RI, United States of America; Public Library of Science, UNITED KINGDOM

## Abstract

**Background:**

Suicide and suicidal behavior during adolescence have been steadily increasing over the past two decades. The preponderance of interventions focuses on crisis intervention, underlying psychiatric disorders, regulating negative affect, and reducing cognitive distortions. However, low positive affectivity may be a mechanism that contributes to adolescent suicidal ideation and behaviors independent of other risk factors. Skills to Enhance Positivity (STEP) is an acceptance-based intervention, designed to increase attention to, and awareness of, positive affect and positive experiences. Results from a pilot RCT demonstrated engagement of the target (positive affect) and a decrease in clinical outcomes (suicidal events; i.e., either a suicide attempt or an emergency intervention for an acute suicidal crisis), providing support to test the clinical effectiveness of STEP in a larger clinical trial with clinical staff implementing the intervention.

**Objective:**

To test the effectiveness of STEP, compared to Enhanced Treatment as Usual (ETAU), in reducing suicidal events and ideation in adolescents admitted to inpatient psychiatric care due to suicide risk. We hypothesize that those randomized to STEP, compared to ETAU, will have lower rates of suicide events, active suicidal ideation (SI), and depressed mood over the 6-month follow-up period. We hypothesize that those randomized to STEP, compared to ETAU, will demonstrate greater improvement in the hypothesized mechanisms of attention to positive affect stimuli and gratitude and satisfaction with life.

**Methods:**

Participants will be randomized to either STEP or ETAU. STEP consists of four in-person sessions focused on psychoeducation regarding positive and negative affect, mindfulness meditation, gratitude, and savoring. Mood monitoring prompts and skill reminders will be sent via text messaging daily for the first month post-discharge and every other day for the following two months. The ETAU condition will receive text-delivered reminders to use a safety plan provided at discharge from the hospital and healthy habits messages, matched in frequency to the STEP group. This trial was registered on 6 August 2021 (ClinicalTrials.gov NCT04994873).

**Results:**

The STEP protocol was approved by the National Institute of Mental Health (NIMH) Data and Safety Monitoring Board on March 4, 2022. The RCT is currently in progress.

**Discussion:**

The STEP protocol is an innovative, adjunctive treatment that has the potential to have positive effects on adolescent suicidal ideation and attempts beyond that found for standard treatment alone.

## Introduction

In the United States, suicide is increasing in the adolescent population [1] and is the second leading cause of death among adolescents in the U.S., with the CDC reporting in 2017 that 17.3% of deaths in youths ages 10–24 were due to suicide [2]. Data from high school students completing the Youth Risk Behavior Surveillance Survey show an increase in the suicide attempt rate as well, from 6.3% in 2009 to 8.9% in 2019 having made one or more attempts in the last year [3]. In 2020, suicide was the occurred at a rate of 10.7% per 100,000 for aged 10–24 [4]. Accordingly, hospital encounters for suicidal adolescents have doubled in the past decade [5]. The period following discharge from a psychiatric hospitalization is a time of increased risk for suicidal behavior [6–8] including deaths by suicide [9]. Our own prospective follow-up studies found 19–20% suicide re-attempt rates by 6 months post-discharge [10, 11]. Inpatient populations are also at risk of treatment disengagement from outpatient services, either through a lapse in the continuity of care or treatment non-adherence [12]. High readmission rates ranging from 30–50% within 30 days of hospitalization have been reported [13, 14]. Thus, intervention efforts that focus on the period immediately following hospitalization may reach the highest-risk adolescents, at the highest-risk time, and reduce suicide attempts and re-admissions.

### Current psychosocial interventions

Psychosocial interventions for suicidal behavior mostly target symptoms of psychiatric illness, often using a cognitive-behavioral therapy (CBT) framework. However, results from two meta-analyses indicate a lack of efficacy in suicide interventions for adolescents [15, 16]. One meta-analysis of CBT to reduce suicidal behaviors identified 6 adolescent and 18 adult studies and found that a significant treatment effect was observed for adult samples but not for adolescents [16]. A more recent meta-analysis which included 9 adolescent studies similarly found that psychotherapy reduced suicide attempts in high-risk adults but not adolescents [15]. One recently published study not represented in these meta-analyses is a multisite Dialectical Behavior Therapy (DBT) study of 173 adolescents [17], which found advantages of DBT over the comparison group at the end of the 6-month treatment period, but not at the 12-month follow-up, in reducing suicide attempts. The DBT protocol, although promising, was highly intensive. The majority of extant studies focus on crisis intervention, psychiatric symptomatology, regulating negative affect, and reducing cognitive distortions. Adjunctive and alternative approaches to reducing suicidal behaviors, particularly in adolescents, are warranted.

### Low positive affect as a potential mechanism of risk for suicidal behavior

Numerous studies suggest that low positive affect is a risk factor for suicidal behavior independent of negative affect and may be another mechanism that leads to suicidal behavior. Low positive affect has been found to prospectively predict time to suicidal events (i.e., either a suicide attempt or an emergency intervention for an acute suicidal crisis) in adolescents over six months of follow-up, even after controlling for a number of other predictors, including depression severity and anhedonia [10]. In a cross-sectional study of over 1000 adolescents, the positive affect experience of gratitude, was inversely associated with suicidal ideation and suicide attempts [18]. The effect of positive affect on suicidal ideation has also been demonstrated in older patients. In a study of 462 primary care patients, age 65 and over, positive affect distinguished people with suicide ideation from those without, even after statistically controlling for the influence of age, gender, depression, negative affect, illness burden, activity, sociability, cognitive functioning and physical functioning [19].

The Broaden and Build theory [20], offers a clear conceptual model for the association between positive affect and suicidal behavior. This model asserts that one function of positive affect may be to broaden attentional scope to help individuals be more open to novel stimuli and social supports, which in turn, broadens and builds psychological and social resources necessary for survival [20–25]. Consistent with this theory, positive affect has been shown to broaden attentional scope [21], motivate thought-action tendencies (i.e., increase behavioral activation) [20], and counter the deleterious effects of negative emotions [26].

Most individuals, particularly those with depressed mood, process emotions through a negativity bias [27], in which individuals pay more attention to and react more strongly to negative stimuli than positive stimuli. Thus, it is likely that positive affect and experiences are frequently being discounted. Understanding the functions of positive affect and negative affect, and intentionally bringing greater attention and awareness to positive affect, may be an important counterweight to this negativity bias, and in turn, may be a mechanism reducing suicide risk.

### Positive psychology interventions

Aside from our pilot studies [28, 29], few Positive Psychology Interventions (PPIs) have specifically been applied to patients with suicidal behavior. Two meta-analyses of PPIs for depression and well-being found that these interventions have small to moderate effects in enhancing well-being and decreasing depressive symptoms and that effects are stronger for individual vs. group formats [30, 31]. However, the vast majority of these interventions were with nonclinical populations, with nearly half of them conducted with college students. Only a handful of studies have been recruited from clinical or hospital settings [32–35]. The few studies conducted with suicidal populations (not included in the meta-analyses) have utilized gratitude exercises and have yielded mixed findings with one study showing favorable effects on suicidal ideation, compared to a food diary, in inpatient adults [36]. Another study, however, found that those randomized to a cognition-focused intervention had significantly greater improvement in depression, suicidal ideation, optimism, and gratitude, compared to the positive psychology intervention [37].

### Skills to Enhance Positivity (STEP)

We developed the Skills to Enhance Positivity (STEP) intervention for adolescents admitted to an inpatient psychiatric unit due to suicide risk. STEP is a multi-modal, adjunctive intervention involving four individual in-person sessions delivered on the inpatient unit, followed by text messaging post-discharge to assess mood and “push” positive affect strategies for up to three months. While STEP borrows heavily from preceding positive psychology interventions such as Seligman’s positive psychotherapy (PPT) [34], it is also distinct. STEP does not address happiness, optimism, or meaning, which may feel unattainable or invalidating to patients in acute distress. To mitigate perceptions of “toxic positivity” [38], STEP is grounded in: psychoeducation on the functions of both negative and positive emotions, the negativity bias that leads to an overemphasis on perceived threats [27], the broaden and build theory of positive emotions [20], and the concept of a positivity ratio [23]. The premise of STEP is that positive emotions and experiences may be easily discounted by the cognitive constriction that often accompanies suicidal ideation and behavior, yet they serve important protective functions. Furthermore, rather than replacing negative with positive, positive emotions can have an additive effect to counterbalance negative emotions. Consequently, specific exercises designed to increase attentional awareness (i.e., psychoeducation on functions of emotions, and specific exercises to cultivate mindfulness, gratitude, and savoring) are practiced, and, in this sense, STEP more closely aligns with acceptance-based approaches to treating depression, which have become increasingly common [39].

STEP was piloted in an open development trial (N = 20) [28] and a pilot RCT (N = 52) [29]; both trials indicated excellent feasibility and acceptability. Preliminary clinical findings of the pilot RCT were promising. Over six months of follow-up, five of 26 participants in the STEP condition (19%) vs. 10 of 26 in the Enhanced Treatment as Usual (ETAU; healthy habits texts) condition (38%) had a suicidal event. Six suicidal events were reported among STEP participants and 13 events were reported among ETAU participants. Thus, those randomized to STEP had 50% fewer suicidal events, in total, and 50% fewer participants engaging in suicidal events, compared to ETAU, the latter corresponding to a medium to large effect size *h* of .43. Those in the STEP condition also saw a more dramatic decrease in active suicidal ideation from baseline compared to their worst week during the follow-up period (STEP 49% vs. ETAU 19% decrease) (Cohen’s d = -0.74). Furthermore, compared to ETAU, parents of STEP participants reported significantly greater improvement in their child’s depression at post-treatment (d = 0.76; 95% CI 0.21–1.30) [29].

Given the strength of our pilot findings, we are conducting a larger, Hybrid Type 1 effectiveness-implementation multi-site study [40] to examine the effectiveness of STEP as an adjunctive intervention to reduce suicide events, active suicidal ideation, and depression, in youth, 12 to 18 years old, admitted to a psychiatric inpatient unit due to suicide risk, over twelve months of post-discharge follow-up. One significant difference between our pilot studies and the current trial is the selection of interventionists. While the initial pilot studies utilized research personnel to deliver STEP, as is appropriate for intervention development studies, the current protocol is delivered by non-doctoral, clinical staff on the unit. The decision was made to use unit staff as the interventionists because decreasing the length of time from pipeline to clinical implementation is paramount. We also created video trainings for STEP interventionists, and brief videos of STEP content which can be used to help the interventionists deliver STEP and also serve as exercise reminders which can be “pushed” remotely after discharge. We will examine the implementation context of STEP in this Hybrid Type 1 trial and to use these data to inform selection of implementation strategies to scale-up, spread, and sustain STEP in additional inpatient units if it proves efficacious.

## Methods

This trial was registered on 6 August 2021 (ClinicalTrials.gov NCT04994873).

### Study design

**Fig 1:**
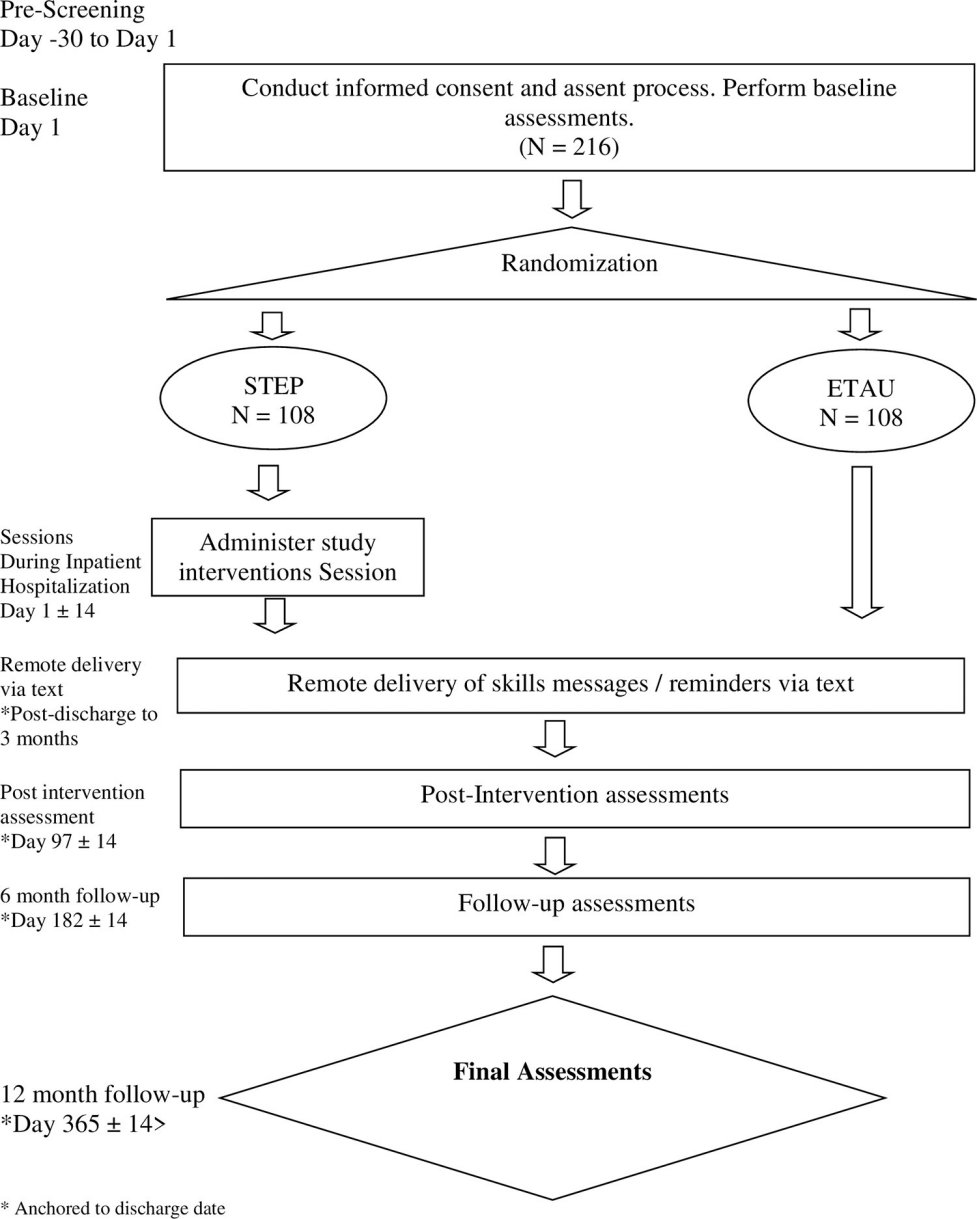
Timeline of interventions and assessments. * Anchored to discharge date.

This study uses a two-group RCT enrolling 216 adolescents to test the primary hypothesis that STEP, delivered to adolescents admitted to an inpatient psychiatric unit, will result in better clinical outcomes than an ETAU condition at the end of the Active Phase of treatment (3-months post-baseline) and at 6- and 12-month follow-up intervals. STEP is an adjunctive, multi-modal intervention that consists of an in-person phase of four sessions, and a remote delivery phase of daily mood ratings and positive affect exercise practice messages delivered via text messaging, which also contains links to videos and mental health resources, for the first month following discharge, followed by tapered messages in the following two months. The ETAU comparison condition is enhanced by healthy habits text messages, as well as reminders to check the participant’s safety plan, matched in frequency to the STEP text messaging protocol. Healthy habits text messages do not overlap with STEP content but are ecologically valid for mental health interventions. Participants in both conditions receive standard care while hospitalized as well as referral to standard mental health care after discharge. (For a timeline of interventions and assessments in each condition, see Fig 1).

Recruitment for this multi-site trial occurs at three adolescent inpatient units from different hospital systems in the Northeast U.S. (first participant recruited on 19 July 2022). The Principal Investigators (PIs) and Site PIs comprise the Steering Committee, and data management is centralized and overseen by a project data coordinator. Data from all sites are stored in REDCap, and when applicable, are double entered to check for accuracy. Oversight for this study, including any modifications to the protocol and safety of study participants, is conducted by a Single IRB (with all other sites ceding) and an NIMH Data and Safety Monitoring Board (DSMB). For continued approval, annual reports are submitted to the IRB and three times a year to the NIMH DSMB.

### Participants

#### Inclusion criteria

1) Adolescents, between the ages of 12–18, hospitalized on an inpatient psychiatric unit due to suicide risk (attempt, severe ideation); 2) Past-month attempts or ideation verified by the Columbia Suicide Severity Rating Scale interview (C-SSRS) [41]; 3) Participants proficient in English, and a parent fluent in either English or Spanish; 4) Participants have access to a smartphone. Parental consent and adolescent assent is obtained from all participants by research personnel; 18-years old participants are consented.

#### Exclusion criteria

The only diagnostic exclusion criteria are psychotic disorders or cognitive deficits that would preclude full understanding of the protocol, intervention, and assessment materials. Determination of mental status as it relates to patients’ ability to fully participate in this study is based on chart review and consultation with unit psychiatrists. Patients who are admitted from, or have a planned discharge to, a residential treatment facility are ineligible. However, those admitted to residential treatment after enrollment in our study can remain in the study.

#### Randomization

Eligible individuals who consent to participate are randomly assigned to either STEP or ETAU. Randomization is stratified by study site, history of suicidal behavior, and whether they identify as a sexual or gender minority, in permuted blocks. The randomization schedule is set up by the study statistician and generated in REDCap. Research assistants, who consent and enroll participants do not have access to the randomization schedule but are able to obtain assignments in real-time, once consent and stratification variable information are recorded. This ensures that research assistants can inform study participants and clinical staff so that they can implement STEP as soon as possible.

### Interventions/Group assignment

Participants in both conditions receive standard inpatient care on each unit. Standard care on the three psychiatric units serving as sites for this study share several commonalities such as: 1) focused psychiatric assessment; 2) a multi-dimensional approach that includes combining individual sessions, group therapies, and medications; 3) inpatient community meetings, occupational therapy support and activities, tutoring; 4) family involvement through meetings and visits; and 5) a secure environment. All units share the goal of stabilizing the child’s behavioral health crisis and, upon discharge, offer recommendations for safety, including a safety plan. All families receive psychoeducation regarding suicidal behavior during their hospitalization, which includes: removing potential suicide means (e.g., guns); being alert to mood changes and suicidal statements; and learning how to access crisis and emergency services. All patients discharged from the inpatient unit are also referred to follow-up psychiatric care (typically an outpatient program, or short-term day treatment program, as well as an appointment with a psychiatrist for medication monitoring), and leave with a copy of their safety plan.

#### Enhanced Treatment as Usual (ETAU)

The comparison condition consists of treatment as usual on the unit which is enhanced at discharge with text messages on healthy habits and reminders to check their safety plan. Participants receive text messages on a daily basis for 30 days. Similar to the STEP condition, and as a means to control for contact time, after 30 days, ETAU participants are placed on a tapered schedule of receiving text messages every other day for an additional two months.

#### Skills to Enhance Positivity (STEP)

STEP consists of an in-person and a remote delivery phase.

#### In-person phase

STEP begins with four individual treatment sessions that include psychoeducation on functions of emotions, mindfulness meditation, savoring, and gratitude. Specific session content is described in Table 1. These practices were specifically selected as they are purported to increase eudemonic positive affect and are consistent with our theoretical framework of accepting the presence and function of negative emotions concomitant with a focus on attending to and cultivating positive emotions as a counterbalance. The psychoeducation session focuses on building rapport and explaining the scientific rationale for our program (e.g., negativity bias, function of positive emotions, counterbalancing negative and positive emotions), to provide context for these practices and obtain participant buy-in. The following sessions which focus on mindfulness mediation, savoring and gratitude respectively, present multiple ways of practicing, asking participants to choose the practice that is most feasible and comfortable for them. Sessions are designed to maximize flexibility such that if not all of the content is delivered in one session, it can be delivered in the following session. When sessions need to be condensed, the brief STEP videos can be utilized to reduce time and burden on the interventionists. Each session lasts approximately 25–30 minutes.

**Table 1:** STEP in-person sessions.

**Session 1: Rapport, Rationale and Psychoeducation** • Brief explanation of program and rationale • Context for admission? • Reasons for living • Protective factors, strengths and resources • Introduce adaptative functions of both PA and NA • Discussion and examples of negativity bias • Introduce simplified version of Broaden and Build theory • Introduce concept of positivity ratio, bank account • Explain objective of daily mood monitoring • Discuss what STEP is not (e.g., toxic • positivity, individual therapy) • Attempt to establish rapport	**Session 2: Mindfulness / Meditation** • Review rationale of increasing attention to positive affect and experiences • What is Mindfulness? What does it do? • Ask about past experiences–likes / dislikes • Discuss that there are different ways of practicing mindfulness • Introduce Mindfulness Meditation grounded in observing breath • Practice a mindfulness exercise (10 deep breaths or other) • Introduce Movement Meditation • Practice a movement meditation (PMR, walking, yoga stretch) • Introduce Compassion/Mantra Meditation • Practice mantra meditation (identify a mantra) • Personalized selection of skills, evaluate feasibility of each type of practice (create buy-in) • Encourage independent practice • Identify barriers to practice in home environment • Troubleshoot
**Session 3: Gratitude** • Review rationale of increasing attention to positive affect and experiences • Review of exercises from previous session including problems or barriers • Why do Gratitude exercises? • Ask about past experiences–likes / dislikes • Different ways of practice • Introduce 3 Good Things • Practice 3 Good Things • Introduce Expression of Gratitude • Envision Expression of Gratitude • Introduce Acts of Kindness–offer examples and discuss impact • Personalized selection of skills, evaluate feasibility of each type of practice (create buy-in) • Encourage independent practice • Identify barriers to practice in home environment	**Session 4: Savoring** • Review rationale of increasing attention to positive affect and experiences • Review of exercises from previous session including problems or barriers • Why do Savoring exercises? • Ask about past experiences–likes / dislikes • Different ways of practice • Introduce Sharing Good Things • Practice Sharing Good Things • Introduce Simple Savoring • Practice Simple Savoring • Introduce Journaling of Positive Events–discuss feasibility • Personalized selection of skills, evaluate feasibility of each type of practice (create buy-in) • Encourage independent practice • Troubleshoot • Review expectations for remote delivery phase via text

#### Remote delivery phase

The remote delivery phase of STEP begins immediately after discharge. STEP participants are asked to complete a set of six mood monitoring questions from the Modified Differential Emotions Scale (mDES) (e.g., “How glad, happy, joyful, do you feel RIGHT NOW? Enter any # from 1 to 5 (1 = not at all, 5 = extremely)”), followed by a request for the participant to select the type of exercise (i.e., mindfulness meditation, gratitude, or savoring) that they would like to practice (“Choose the type of message you would like to receive right now: 1 = mindfulness; 2 = gratitude; 3 = savoring”). In response, the participant receives a corresponding exercise to practice (e.g., “share something positive with a friend or family member”). Participants are also asked if they had practiced a positive affect exercise the preceding day. Periodically, STEP participants are sent exercise reminders regardless of their engagement with text prompts, thus ensuring that all participants randomized to STEP continue to receive the intervention. No questions about suicidal ideation are asked; nonetheless, a message is displayed to let users know that no one is reading their responses and that if they are experiencing a mental health crisis, to contact their provider and/or Emergency Department. The same message is displayed to the ETAU group. Participants assigned to ETAU do not receive mood monitoring questions as mood monitoring is conceptualized as part of the intervention. Like the ETAU condition, after the 30 days of daily practice reminders, participants are placed on a tapered schedule of every other day for an additional two months.

### Therapist training

Therapists for this study are clinical staff based on the unit (e.g., milieu therapists, social work interns) who receive training to administer STEP. Training materials include a written treatment manual and five audio- and video-recordings describing the premise of STEP and illustrating each of the sessions. To enhance feasibility, training is self-paced and available on-demand. After reviewing the training materials, interventionists are asked to record role plays that are reviewed by trained interventionists, and then feedback is provided. Periodic booster trainings are conducted as needed.

### Treatment integrity

As STEP is being deployed in a clinical setting by on-duty clinicians, recording actual sessions is not feasible. Thus, we developed self-administered adherence checklists that cover the most important elements of the intervention for each of the four sessions. Interventionists are asked to also rate their audio-recorded role plays to further reinforce training. These ratings will be compared against the ratings performed by a STEP trainer, and the interventionist will receive feedback on any differences in ratings; adherence over 80% will be considered acceptable. After the first three study participants, interventionists will be asked to do another rated role play to ensure that they are administering STEP as intended. Treatment integrity assessments also provide implementation outcome data for the real-world delivery of STEP in a future trial, to determine the extent to which frontline clinicians are able to deliver STEP with fidelity [42], and without a voltage drop or program drift, two of the main concerns with future sustainability of any evidence-based intervention in usual care settings [43].

### Assessments

**Fig 2:**
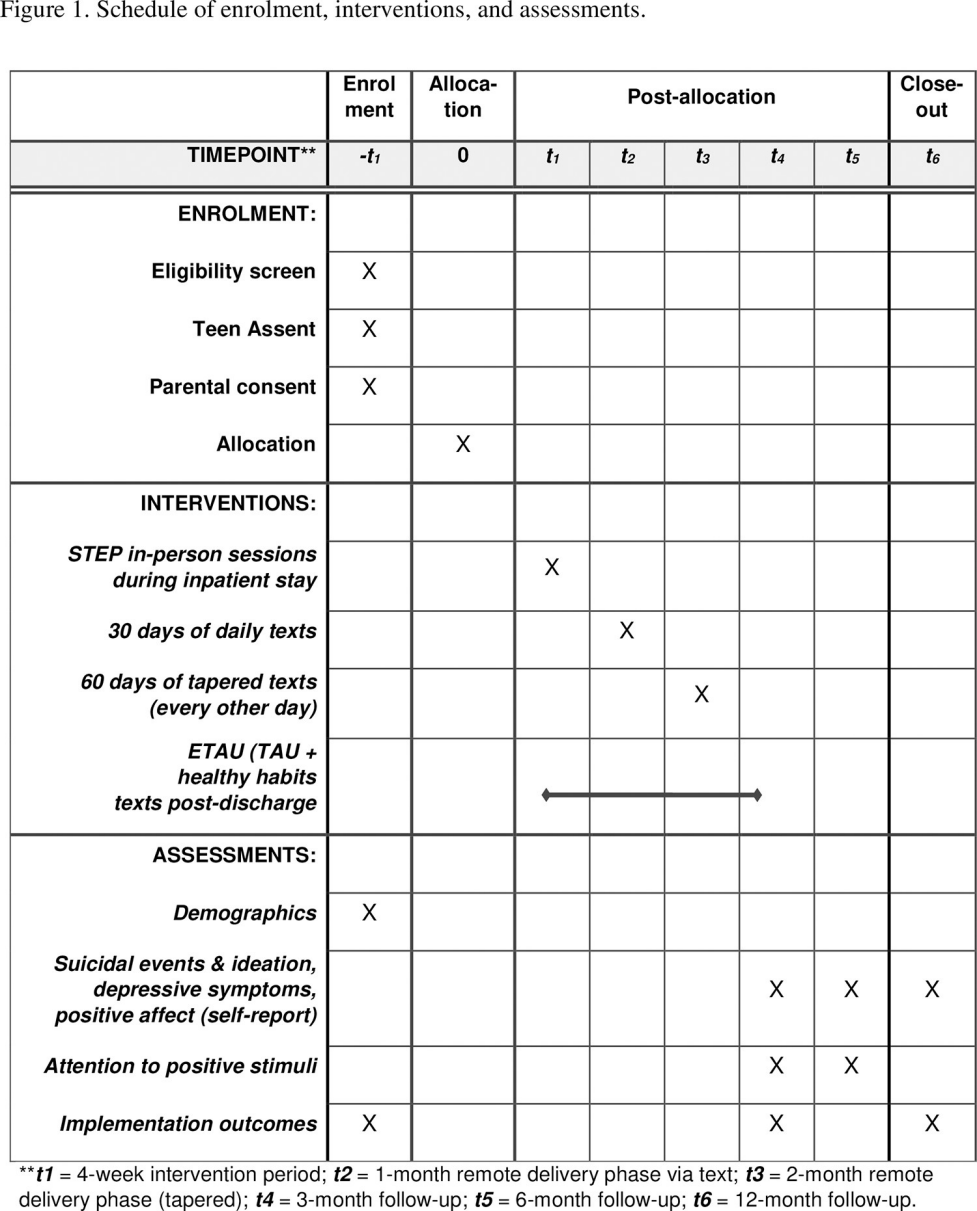
Schedule of enrolment, interventions, and assessments. **t1 = 4-week intervention period; *t2* = 1-month remote delivery phase via text; *t3* = 2-month remote delivery phase (tapered); *t4* = 3-month follow-up; *t5* = 6-month follow-up; *t6* = 12-month follow-up.

The assessment battery is administered at baseline (prior to randomization) by the research personnel. At the end of the active intervention phase (3 months post-baseline), and at the 6- and 12-month follow-up intervals, assessments are conducted by a masked outcome assessor who does not know participants’ condition assignment. (See Fig 2 for a schedule of assessments at each timepoint).

### Measures

#### Background variables

Routine demographic variables are collected that assess current living situation, legal involvement, history of abuse, family psychiatric history and treatment history. Self-report forms will be used to ask about racial, ethnic, sexual and gender identity.

#### Primary outcomes

***Suicidal events*** are operationalized as a composite score of suicide attempts and inpatient hospital admissions or ED visits due to suicide risk. This is assessed with the Columbia-Suicide Severity Rating Scale [41] and interview questions regarding treatments received during the study trial. The C-SSRS assesses the range of suicidal behavior including preparatory acts, aborted and interrupted attempts, passive and active suicidal ideation, as well as intensity of ideation. At follow-up, suicidality since the last assessment is assessed. Interviews will be administered at baseline, 3-, 6-, and 12-month follow-up, with the 6-month follow-up as our primary outcome.

***Suicidal Ideation*** is also assessed using the C-SSRS. Specifically, we assess active suicidal ideation, operationalized by a score of 3 or higher on the suicide ideation questions of C-SSRS, using their highest level of ideation during the worst week of the follow-up interval. We also obtain weekly ratings of suicidal ideation using the methodology of the Longitudinal Interval Follow-Up Evaluation (LIFE) [44], at the 3-, 6-, and 12 month follow-up. The LIFE format asks participants to rate their level of SI in the preceding interval on a 6-point psychiatric status rating (PSR) scale.

***Depressive symptoms*** is assessed by the Beck Depression Inventory (BDI-II) [45], administered to both adolescent and parent (about their child). The BDI-II is a widely used 21-item measure with excellent psychometric properties [46]. Cronbach’s α in the pilot sample was 0.90 in adolescents and 0.89 in adults. Assessments are administered at baseline, 3-, 6-, and 12-months f/u.

#### Mediators

*Attention to positive stimuli*. Adolescents’ attentional biases for emotional stimuli are assessed using a Tobii Pro Nano eye-tracker while they complete a modified dot probe task (cf. [47]. Stimuli consist of pairs of facial expressions that contain one emotional (happy, or sad) and one neutral photograph from the same actor taken from a standardized stimulus set [48]. Photographs from each actor (16 males and 16 females) were used to create happy-neutral and sad-neutral stimulus pairs (64 pairs total), with each pair presented twice (128 trials total). Emotional and neutral stimuli appear with equal frequency on the left and right side of the screen. Stimuli are presented for 1,000 ms followed by a probe (E or F) replacing one of the pictures. Participants are asked to indicate the identity of the probe (E or F) as quickly as possible using the keyboard. Variables of interest are location and latency of first fixation and overall gaze duration to each stimulus type.

We also assess attention to PA using the IPANAT [49], an indirect assessment of automatic activation of affective representations that utilizes artificial words paired with positive and negative words. Factor analyses of ratings yield two independent factors interpreted as positive and negative. A single score of implicit affect (positive, negative) reflects both state and trait variance. The IPANAT has demonstrated strong convergent and discriminant validity, and adequate internal consistency and test-retest reliability [49]. These proposed tasks are administered at baseline, 3-, and 6-month follow-ups.

*Self-report of positive affect*. Self-report ratings of positive and negative affect are assessed at baseline, 3-, 6-, and 12-month follow-up, through the Modified Differential Emotions Scale (mDES). The 19-item mDES assesses short-term state positive and negative emotions, along a 5-point Likert-type scale. Each item consists of three related terms to describe a particular discrete emotion (e.g., glad, happy, joyful). Participants are asked to rate each set of emotions based on how they were feeling “right now.” The Positive Emotions subscale is a composite of 10 items, while the Negative Emotions subscale is a composite of 9 items. The scale has psychometric support [24] and in the pilot sample, Cronbach’s α was = .86 and .92 for the positive and negative emotions subscales, respectively. To obtain subjective indices of eudemonic (i.e., sustainable) positive affect, we administer the Gratitude Questionnaire (GQ6) [50, 51] and the Satisfaction with Life Scale (SWLS) [52]. Both scales use a 7-point Likert response format and will be administered only to the adolescent.

#### Implementation outcomes

Implementation evaluation is based on Proctor et al.’s [42] categorization of implementation outcomes. This study measures 1) acceptability, or the extent to which adopting STEP is agreeable, palatable, or satisfactory among key stakeholders, 2) appropriateness, the perceived fit, relevance or compatibility of STEP for a given setting, provider or family; and 3) feasibility, the extent to which STEP can be successfully used or carried out within a given setting. These will be examined using the Acceptability of Intervention Measure (AIM), Intervention Appropriateness Measure (IAM), and the Feasibility of Intervention Measure (FIM), after all post-treatment data have been collected [53]. Each are 4-item scales that have demonstrated good psychometric properties including good test-retest reliability and discriminant validity [53].

### Data analyses

Data from all randomized participants will be included in analyses (intent-to-treat). Preliminary analyses will include descriptive statistics to examine the distributional and psychometric properties of the variables (e.g., normality, internal consistency). Variables will be transformed to better approximate normality if this is necessary given the observed distribution and the assumptions of the analysis model. We will also examine post-inclusion attrition by comparing study completers to dropouts on sociodemographic, baseline data, and psychotropic medication usage to determine if they differ systematically. To accommodate attrition and other sources of missingness, we will utilize the method of multiple imputation by chained equations. We will examine the success of the stratified randomization by examining between-group differences at baseline. Preliminary analyses may also include analyses of adverse events, the progress of recruitment and retention, and data processing and quality markers as the study progresses. Daily mood monitoring data is not included in outcome analyses because it is conceptualized as part of this adjunctive intervention and only collected in one condition.

To examine the effectiveness of STEP in reducing suicidal events, active SI, and depression at 3- and 6-month follow-up (primary) and 12 month follow-up (secondary), we will use a repeated measures design and a multilevel or mixed effect modeling framework for continuous outcomes (e.g., proportion of weeks with active SI, depression) and a mixed effect logistic regression model will be used for binary outcomes (e.g., suicide events). For all hypotheses, we will treat the individual as the random effect. Treatment effects will be estimated as the weighted average of the differences between ETAU and STEP groups over all follow-up times, adjusting for follow-up time.

#### Sample size and statistical power considerations

We determined minimal detectable effect sizes using Monte Carlo methods. This involved generating 1,001 simulated data sets representing our hypothesized effects, and our assumptions of a 10% loss by 3-month and another 10% loss by 6-month follow-up. We assumed repeated measures of depressive symptoms and the propensity to active suicidal ideation and suicidal events were all correlated at *r* = .5. Across the simulated data sets, we compute power as the proportion of replicates returning a significant effect in the hypothesized direction. For each of our outcomes, we will have sufficient power to detect small-to-medium Hedges’ *g* effect sizes (.46, .31, and 35, respectively).

### Ethics approval and consent to participate

This study was approved by the Butler Hospital Institutional Review Board (Study # 2104–002) serving as the Single IRB for the other institutions in this multisite study including Boston Children’s Hospital, Bradley Hospital, and Brown University. All study materials and protocols have been ethically reviewed, both in English and Spanish where appropriate. All substantial amendments will be reviewed by the Butler Hospital Institutional Review Board. Informed consent for the parents and informed assent for the children will be obtained from all participants.

## Discussion

STEP is a program that translates findings from social psychology into clinical interventions in a delivery that is feasible and accessible for high-risk adolescents. Our proposed treatment represents a paradigm shift from a traditional risk or deficit-based model to an enhancement model for reducing suicidal behaviors. Most interventions for suicidal behavior target specific disorders (e.g., depression) or risk vulnerabilities (e.g., substance use), and prioritize discussion of risk behavior over positive experiences. In contrast, STEP is transdiagnostic and targets positive affect as an ancillary mechanism to counterbalance the negativity bias observed in depressed and suicidal individuals. Our intervention is premised on the Broaden and Build theory of positive affect and the importance of having a healthy ratio of positive to negative affect, thus implicitly integrating both types of affective experience. This approach is also consistent with more recent acceptance-based frameworks of psychotherapy, and distinct from other positive psychology interventions (e.g., which focus on change-based orientation towards happiness, optimism, fulfillment). Despite the emergence and proliferation of positive affect exercises in popular culture, there is a dearth of adequately powered clinical trials that test these interventions. Ours is the first study of a positive affect-based adjunctive intervention developed to decrease suicidal thoughts and behaviors in adolescents.

STEP was designed to maximize at-home practice, increasing the likelihood that they will be used on a consistent basis. We do this by pairing in-person sessions with remote delivery. This multi-modal approach allows for personal engagement and also increases the utilization of digitally delivered interventions, which have been demonstrated to have a steep drop off in utilization when delivered alone [54]. The use of basic technology (text messages) to engage high-risk youth who may encounter barriers to subsequent care also balances the need to achieve engagement with a vulnerable population and maintenance of treatment objectives through low-cost, broadly accessible means.

Implementing a new clinical intervention into busy clinical settings, such as an inpatient unit, has its challenges. A Hybrid Type 1 effectiveness-implementation study allows for the testing of the effectiveness of STEP while simultaneously gathering information on the implementation outcomes, thereby providing data on whether STEP can be delivered as intended in a real-world setting. STEP is comprised mainly of psychoeducation and exercise practice, is easily deliverable by frontline milieu staff, or psychology or social work trainees at academic teaching hospitals. However, the recent shortage of mental health workers particularly in inpatient and residential settings, while presenting additional challenges, also underscores the importance of developing short-term low-cost hybrid interventions that are easy to train and deploy.

## Supporting information

S1 File SPIRIT checklist. (PDF)

S2 File IRB-approved protocol. (DOCX)
