# Supplementary material for: Skills to Enhance Positivity in adolescents at risk for suicide: Protocol for a randomized controlled trial
Source: PLoS One. 2023 Oct 20;18(10):e0287285. doi: 10.1371/journal.pone.0287285 (PMC10588868; doi:10.1371/journal.pone.0287285)
Supplement: S2 File — (DOCX) [file pone.0287285.s002.docx]

**Skills to Enhance Positivity (STEP)**

**Protocol Number: <1722662-2>**

**National Clinical Trial (NCT) Identified Number:** NCT04994873

**Principal Investigator:** **Anthony Spirito, Ph.D.; Shirley Yen, Ph.D.**

**Sponsor: Brown University**

**Grant Title: Skills to Enhance Positivity in Adolescents at Risk for Suicide**

**Grant Number: MH123556**

**Funded by: National Institute of Mental Health**

**03/17/2023**

Table of Contents

[INVESTIGATOR’S SIGNATURE 2](#_Toc127455061)

[1 PROTOCOL SUMMARY 3](#_Toc127455062)

[1.1 Synopsis 3](#_Toc127455063)

[1.2 Schema 4](#_Toc127455064)

[1.3 Schedule of Activities 5](#_Toc127455065)

[2 INTRODUCTION 6](#_Toc127455066)

[2.1 Study Rationale 6](#_Toc127455067)

[2.2 Background 6](#_Toc127455068)

[2.3 Risk/Benefit Assessment 9](#_Toc127455069)

[2.3.1 Known Potential Risks 9](#_Toc127455070)

[2.3.2 Known Potential Benefits 11](#_Toc127455071)

[2.3.3 Assessment of Potential Risks and Benefits 11](#_Toc127455072)

[3 OBJECTIVES AND ENDPOINTS 12](#_Toc127455073)

[4 STUDY DESIGN 13](#_Toc127455074)

[4.1 Overall Design 13](#_Toc127455075)

[4.2 Scientific Rationale for Study Design 14](#_Toc127455076)

[4.3 Justification for Intervention 14](#_Toc127455077)

[4.4 End-of-Study Definition 14](#_Toc127455078)

[5 STUDY POPULATION 14](#_Toc127455079)

[5.1 Inclusion Criteria 15](#_Toc127455080)

[5.2 Exclusion Criteria 15](#_Toc127455081)

[5.3 Screen Failures 15](#_Toc127455082)

[5.4 Strategies for Recruitment and Retention 15](#_Toc127455083)

[6 STUDY INTERVENTION(S) OR EXPERIMENTAL MANIPULATION(S) 17](#_Toc127455084)

[6.1 Study Intervention(s) or Experimental Manipulation(s) Administration 18](#_Toc127455085)

[6.1.1 Study Intervention or Experimental Manipulation Description 18](#_Toc127455086)

[6.1.2 Administration and/or Dosing 18](#_Toc127455087)

[6.2 Fidelity 19](#_Toc127455088)

[6.2.1 Interventionist Training and Tracking 20](#_Toc127455089)

[6.3 Measures to Minimize Bias: Randomization and Blinding 20](#_Toc127455090)

[6.4 Concomitant Therapy 21](#_Toc127455091)

[6.4.1 Rescue Therapy 21](#_Toc127455092)

[7 STUDY INTERVENTION/EXPERIMENTAL MANIPULATION DISCONTINUATION AND PARTICIPANT DISCONTINUATION/WITHDRAWAL 21](#_Toc127455093)

[7.1 Discontinuation of Study Intervention/Experimental Manipulation 21](#_Toc127455094)

[7.2 Participant Discontinuation/Withdrawal from the Study 21](#_Toc127455095)

[7.3 Lost to Follow-Up 22](#_Toc127455096)

[8 STUDY ASSESSMENTS AND PROCEDURES 22](#_Toc127455097)

[8.1 Endpoint and Other Non-Safety Assessments 22](#_Toc127455098)

[8.2 Safety Assessments 24](#_Toc127455099)

[8.3 Adverse Events and Serious Adverse Events 25](#_Toc127455100)

[8.3.1 Definition of Adverse Events 25](#_Toc127455101)

[8.3.2 Definition of Serious Adverse Events 25](#_Toc127455102)

[8.3.3 Time Period and Frequency for Event Assessment and Follow-Up 26](#_Toc127455103)

[8.3.4 Adverse Event Reporting 26](#_Toc127455104)

[8.3.5 Serious Adverse Event Reporting 27](#_Toc127455105)

[8.4 Unanticipated Problems 27](#_Toc127455106)

[8.4.1 Definition of Unanticipated Problems 27](#_Toc127455107)

[8.4.2 Unanticipated Problems Reporting 27](#_Toc127455108)

[9 STATISTICAL CONSIDERATIONS 28](#_Toc127455109)

[10 SUPPORTING DOCUMENTATION AND OPERATIONAL CONSIDERATIONS 31](#_Toc127455110)

[10.1 Regulatory, Ethical, and Study Oversight Considerations 31](#_Toc127455111)

[10.1.1 Informed Consent Process 31](#_Toc127455112)

[10.1.2 Study Discontinuation and Closure 32](#_Toc127455113)

[10.1.3 Confidentiality and Privacy 32](#_Toc127455114)

[10.1.4 Future Use of Stored Specimens and Data 33](#_Toc127455115)

[10.1.5 Key Roles and Study Governance 33](#_Toc127455116)

[10.1.6 Safety Oversight 34](#_Toc127455117)

[10.1.7 Clinical Monitoring 34](#_Toc127455118)

[10.1.8 Quality Assurance and Quality Control 34](#_Toc127455119)

[10.1.9 Data Handling and Record Keeping 35](#_Toc127455120)

[10.1.10 Protocol Deviations 35](#_Toc127455121)

[10.1.11 Publication and Data Sharing Policy 36](#_Toc127455122)

[10.1.12 Conflict of Interest Policy 36](#_Toc127455123)

[10.2 Abbreviations and Special Terms 36](#_Toc127455124)

[10.3 Protocol Amendment History 36](#_Toc127455125)

[11 REFERENCES 38](#_Toc127455126)

STATEMENT OF COMPLIANCE

1. The trial will be carried out in accordance with International Council on Harmonization Good Clinical Practice (ICH GCP) and the following:

- United States (US) Code of Federal Regulations (CFR) applicable to clinical studies (45 CFR Part 46, 21 CFR Part 50, 21 CFR Part 56, 21 CFR Part 312, and/or 21 CFR Part 812).

National Institutes of Health (NIH)-funded investigators and clinical trial site staff who are responsible for the conduct, management, or oversight of NIH-funded clinical trials have completed Human Subjects Protection and ICH GCP Training.

The protocol, informed consent form(s), recruitment materials, and all participant materials will be submitted to the IRB for review and approval. Approval of both the protocol and the consent form(s) must be obtained before any participant is consented. Any amendment to the protocol will require review and approval by the IRB before the changes are implemented to the study. All changes to the consent form(s) will be IRB approved; a determination will be made regarding whether a new consent needs to be obtained from participants who provided consent, using a previously approved consent form.

# INVESTIGATOR’S SIGNATURE

The signature below constitutes the approval of this protocol and provides the necessary assurances that this study will be conducted according to all stipulations of the protocol, including all statements regarding confidentiality, and according to local legal and regulatory requirements and applicable US federal regulations and ICH guidelines.

Principal Investigator or Clinical Site Investigator:

| Signed: | Anthony Spirito, PhD | Date: | 02-09-2022 |
| --- | --- | --- | --- |
|  | Name: Anthony Spirito, PhD | | |
|  | Title: Professor/MPI | | |

Investigator Contact Information
Affiliation: Department of Psychiatry and Human Behavior, Brown University

Address: Box G-BH, Providence, RI 02912

Telephone: 401-444-1929

Email: [Anthony_Spirito@Brown.edu](mailto:Anthony_Spirito@Brown.edu)

[For multi-site studies, the protocol should be signed by the clinical site investigator who is responsible for the day to day study implementation at his/her specific clinical site.]

| Signed: | Shirley Yen, PhD | Date: | 02-09-2022 |
| --- | --- | --- | --- |
|  | Name: Shirley Yen, PhD | | |
|  | Title: Associate Professor, MPI | | |
|  | Affiliation: Beth Israel Deaconess Medical Center, Harvard Medical School | | |

# PROTOCOL SUMMARY

## Synopsis

| **Title:** | Skills to Enhance Positivity in Adolescents at Risk for Suicide |
| --- | --- |
| **Grant Number:** | MH123556-01A1 |
| **Study Description:** | The purpose of this study is to examine the effectiveness of the Skills to Enhance Positivity (STEP) intervention in reducing suicidal behaviors in adolescents who have been admitted to an inpatient psychiatric unit due to suicide risk. |
| **Objectives:** | Primary Objective: Examine the effectiveness of STEP in reducing suicidal events (suicide attempt or emergency intervention to intercede attempt), active SI (with intent or plan), and depression at 6-month follow-up (primary endpoint outcomes) and suicidal events at 12-month follow-up (distal, endpoint outcome).  Secondary Objective: Examine engagement of the hypothesized mechanisms (attention to positive and negative affect) at the 3- and 6-month follow-up.  Tertiary Objective: Examine whether hypothesized mechanisms mediate reduction of suicidal events and ideation. |
| **Endpoints:** | Primary Endpoint: Suicidal events, active suicidal ideation, and depression at 6 month follow-up.  Distal Primary Endpoint: Suicidal events at 12 month follow-up.  Secondary Endpoint: Attention to Positive Affect and Negative Affect at 6 month follow-up. |
| **Study Population:** | Adolescents, regardless of sex, gender, race, and ethnicity, between the ages of 12-18, who have been hospitalized due to suicide risk (i.e., a suicide attempt or suicidal ideation) will be recruited for study participation from three adolescent units at Boston Children’s Hospital (Boston, MA), Butler Hospital (Providence, RI), & Bradley Hospital (Providence, RI). Target recruitment is 216. |
|  |  |
| **Phase or Stage:** | Phase III |
| **Description of Sites/Facilities Enrolling Participants:** | Three sites, all in the United States, will be enrolling participants: 1) the adolescent inpatient unit at Boston Children’s Hospital; 2) the adolescent unit of Butler Hospital; and 3) the adolescent inpatient unit at Bradley Hospital |
| **Description of Study Intervention/Experimental Manipulation:** | The study intervention, Skills to Enhance Positivity (STEP), is an adjunctive psychosocial intervention that involves 4 in-person session (4 individual) during a participant’s inpatient admission, followed by three months of text or email messaging for mood monitoring and skills delivery. The sessions and skills delivered via the messaging focus on the functions of positive affect and ways to bring greater attention to and awareness of positive emotions and experiences on a daily basis. |
| **Study Duration:** | 36 months |
| **Participant Duration:** | 12 months |

## Schema

***Flow Diagram for Randomized Clinical Trial***

Pre-screen potential participants by inclusion and exclusion criteria via chart review.

Pre-Screening

Day -30 to

Day 1

Conduct informed consent and assent process. Perform baseline assessments.

(N = 216)

Refer to **Section 1.3, Schedule of Activities**>

Baseline

Day 1

Randomize

Administer study interventions Session 1-4; Provide parent infographic

Sessions

During Inpatient

Hospitalization

Day 1 ± 14

Remote delivery

Remote delivery of skills messages / reminders via text or email

via text or email

*Post-discharge to

3 months

Post-Intervention assessments

Post intervention

assessment

*Day 97 ± 14

Follow-up assessments

6 month follow-up

*Day 182 ± 14

**Final Assessments**

Refer to **Section 1.3, Schedule of Activities**>

12 month follow-up

*Day 365 ± 14>

* Anchored to discharge date

## Schedule of Activities

| **TABLE OF ASSESSMENTS** |  |  |  |  |  |  |
| --- | --- | --- | --- | --- | --- | --- |
| **Instrument** | **Admin to:** | **Type** | **Base** | **3M** | **6M** | **12M** |
| **Demographics** | A, G | Interview | X |  |  |  |
| **Suicidal and Self-Harm Behaviors:** |  |  |  |  |  |  |
| Columbia Suicide Severity Rating Scale (C-SSRS) | A, G | Interview | X | X | X | X |
| Suicide Ideation Questionnaire (SIQ) | A | SR | X | X | X |  |
| Suicidal Ideation Psychiatric Rating Scale – Longitudinal Follow-up Evaluation (LIFE-SI-PSR) | A | Interview | X | X | X |  |
| Functional Assessment of Self-Mutilation (FASM) | A | SR | X | X | X |  |
| **Diagnostic / Symptom Rating Scales:** |  |  |  |  |  |  |
| Beck Depression Inventory (BDI-II) | A, G | SR | X | X | X |  |
| Alcohol Use Disorder Identification Test (AUDIT) | A, G | SR | X | X | X |  |
| Drug Use Disorder Identification Test (DUDIT) | A, G | SR | X | X | X |  |
| Borderline Evaluation of Severity Over Time (BEST) | A, G | SR | X | X | X |  |
| Columbia Impairment Scale (CIS) | A, G | SR | X | X | X |  |
| **Treatment Targets of STEP:** |  |  |  |  |  |  |
| Attention Dot Probe Positive and Negative Stimuli | A | Task | X | X | X |  |
| Implicit Positive and Negative Affect Test | A | Task | X | X | X |  |
| Modified Differential Emotions Scale (mDES) | A | SR | X | X | X |  |
| Satisfaction with Life Scale (SWLS) | A | SR | X | X | X |  |
| Gratitude Questionnaire (GQ-6) | A | SR | X | X | X |  |
| **Ancillary Treatment Received:** |  |  |  |  |  |  |
| Treatment History Interview | A, G | Interview | X | X | X |  |
| Medication Compliance Questionnaire | A, G | Interview | X | X | X |  |
| **Implementation:** |  |  |  |  |  |  |
| Client Satisfaction Questionnaire | A, G | SR |  | A, G |  |  |
| Acceptability of Intervention Measure (AIM) | A, G | SR |  | A,G |  |  |
| Intervention Appropriateness Measure (IAM) | A, G | SR |  | A,G |  |  |
| Feasibility of Intervention Measure (FIM) | A, G, P (at different timepoints) | SR | P | A, G |  | P |
| Qualitative Exit Interviews | A, G, P (at different timepoints) | Interview | P | A, G |  | P |
| Evidenced Based Practice Attitude Scale (EBPAS) | P | SR | P |  |  | P |
| Implementation Climate Scale | P | SR | P |  |  | P |
| Implementation Readiness Scale | P | SR | P |  |  | P |

***X denotes administration of that instrument***

**A = Adolescent; P = Provider; G = Guardian; SR = Self-Report**

**Guardians will be asked to assess and report on adolescents (not self-report) using the instruments listed above****

# INTRODUCTION

## Study Rationale

Suicide and suicidal behavior in adolescents have been steadily increasing over the past two decades. Unfortunately, reviews of published randomized controlled trials (RCTs) for adolescent suicidality conclude that treatments to date have been minimally efficacious, particularly when compared to adult trials. The preponderance of interventions focus on crisis intervention, underling psychiatric disorders, regulating negative affect, and reducing cognitive distortions. However, our pilot work and other recent data suggest the importance of considering how *low positive affectivity* *may be a mechanism that contributes to suicidal behaviors independent of other risk factors.* Therefore, we developed an intervention, *Skills to Enhance Positivity* (STEP; MH R34101272), premised on the Broaden and Build theory of positive affect, to increase attention to, and awareness of positive affect and experiences. Results from our pilot RCT (N=52) with inpatient suicidal adolescents found that compared to a Healthy Habits / Enhanced Treatment as Usual (ETAU) condition, those randomized to STEP had 50% fewer individuals reporting a suicide event, 50% fewer suicidal events overall, and a larger decrease in participants reporting active suicidal ideation (SI) over follow up (f/u) (49% STEP vs. 19% ETAU). STEP also appears to have engaged the target mechanism as STEP participants had faster reaction times to positive probes on an attentional bias task compared to ETAU.

These promising results in which we were able to demonstrate engagement of the target (positive affect) and a decrease in clinical outcomes (suicidal events) suggest the need to test the clinical effectiveness of STEP by having clinical staff implement the intervention. Furthermore, recognizing the need to speed translation, we propose a Hybrid Type I Effectiveness-Implementation design. Specifically, we propose to test the effectiveness of STEP in reducing suicidal events and ideation in 216 adolescents, admitted to inpatient psychiatric care due to suicide risk. Participants will be randomized to either STEP or ETAU. STEP involves 4 in-person sessions focused on psychoeducation regarding positive and negative affect, mindfulness meditation, gratitude, and savoring. Parents will also be provided with a handout containing study contact information and key information on how parents/guardians can best support their children throughout the study. 4 videos with content covered in the treatment manual will be used to supplement the skills taught during the 4 in-person STEP sessions. Mood monitoring prompts and skill reminders will be sent daily for the first month post-discharge and three times a week for the following two months. The ETAU condition will receive text or email reminders to visit their safety plan or practice a Healthy Habit, matched in frequency to the STEP group. Effectiveness aspects of the design include using clinical staff as interventionists and having very few exclusion criteria.

The public health significance of this application is evident as it addresses the Healthy People 2020 Objective MHMD HP 2020- 2: “Reduce suicide attempts by adolescents.” This application also strongly aligns with NIMH’s interest in improving post-acute care and optimizing longer term outcomes (PAR-18-430), as well as the NIMH 2020 Strategic Plan for Research, specifically objective 3.3. “test interventions for effectiveness in community practice settings” and 3.3.c “enhancing the practical relevance of effectiveness research via deployment-focused hybrid effectiveness-implementation studies”.

## Background

**Scope of Problem.** Suicide and suicidal behavior (SB) during adolescence has been increasing over the past two decades. Suicide is the second leading cause of death among adolescents in the U.S., with the CDC reporting in 2017 that 17.3% of deaths in youths ages 10-24 were due to suicide (1). The trend is particularly alarming for girls who have experienced a three-fold increase in suicide deaths since 1999 (2). Data from high school students completing the Youth Risk Behavior Surveillance Survey show an increase in the suicide attempt rate as well, from 6.3% in 2009 to 8.6% in 2015 (3).

Accordingly, hospital encounters for suicidal adolescents have doubled in the past decade (4). The period following discharge from a psychiatric hospitalization is a time of increased risk for suicidal behavior (5-7) including suicide deaths (8). Our own prospective follow-up studies found 19% and 20% suicide re-attempt rates by 6 months post-discharge (9, 10). Inpatient populations are also at risk of treatment disengagement from outpatient services, either through a lapse in the continuity of care or treatment non-adherence (11). High re-admission rates ranging from 30-50% within 30 days of hospitalization have been reported (12, 13). Thus, intervention efforts that focus on the period immediately following hospitalization may efficiently reach the highest-risk adolescents, at the highest-risk time, and reduce healthcare costs incurred by re-admissions.

**Current Psychosocial Interventions.** Psychosocial interventions for suicidal behavior mostly target symptoms of psychiatric illness, often using a cognitive-behavioral therapy (CBT) framework. However, results from two meta-analyses indicate a lack of efficacy in suicide interventions for adolescents (14, 15). One meta-analysis of CBT to reduce SB identified 6 adolescent and 18 adult studies and found that a significant treatment effect was observed for adult samples but *not* for adolescents (15). A more recent meta-analyses which included 9 adolescent studies similarly found that psychotherapy reduced suicide attempts in high-risk adults but not adolescents (14). The study that showed the strongest effect in this meta-analyses, was designed for youth with comorbid substance use and suicidality and conducted by our group (16). Unfortunately, a larger trial of this same intervention, which did not require youth report substance use, did not yield significant between-group effects on suicide related outcomes (10). One recently published study not represented in these meta-analyses is a multisite DBT study of 173 adolescents (17), which found advantages of Dialectical Behavior Therapy (DBT) over the comparison group at the end of the 6 month treatment period but not at the 12-month f/u on SA. This study, although promising, was highly intensive, as have been almost all studies to date*.* The majority of studies reviewed here and represented in the meta-analyses, primarily focus on crisis intervention, psychiatric symptomatology, regulating negative affect, and reducing cognitive distortions. Alternative approaches to reducing suicidal behaviors, particularly in adolescents, are warranted.

**Low Positive Affect (PA).** Our pilot work and other recent data suggest the importance of considering how low PA contributes to suicide risk. In a study of psychiatrically hospitalized suicidal adolescents conducted by the MPIs, low PA prospectively predicted time to suicidal events, i.e., either a suicide attempt or an emergency intervention for an acute suicidal crisis, in adolescents over 6 months of f/u. This effect remained significant after controlling for a number of other predictors, including depression severity and anhedonia (Hazard Ratio = 1.82; p<.05) (9). In a cross-sectional study of over 1000 adolescents, gratitude was inversely associated with suicidal ideation (SI) and suicide attempts (18). The effect of PA on SI has also been demonstrated in older patients. In a study of 462 primary care patients, age 65 and over, PA distinguished suicide ideators from non-ideators, after controlling for age, gender, depression, negative affect (NA), illness burden, activity, sociability, cognitive functioning and physical functioning (19). These studies suggest that low PA is a risk factor for SB independent of negative affect (NA) and may be another mechanism that leads to SB.

The *Broaden and Build theory* offers a clear conceptual model for the association between PA and SB. This model asserts that one function of PA may be to broaden attentional scope to help individuals be more open to novel stimuli and social supports, which in turn, broadens and builds psychological and social resources necessary for survival (20-25). PA has been shown to broaden attentional scope (21), motivate thought-action tendencies (i.e. increase behavioral activation) (20), and counter the deleterious effects of negative emotions (26). Drs. Fredrickson and Joiner examined the reciprocity of influence between PA and “broad-minded coping” measured by items such as “think of different ways to deal with the problem,” and “try to step back from the situation and be more effective” (23). They assessed positive and negative emotions at two time points 5 weeks apart and found that those who experienced more positive emotions had higher increases in broad-minded coping. In turn these skills predicted increased positive emotions over time.

Most individuals, particularly those who are depressed, process emotions through a negativity bias (27), in which individuals pay more attention to and react more strongly to negative stimuli than positive stimuli; thus, it is likely that positive affect and experiences are frequently being discounted. Understanding the functions of PA and NA, and intentionally bringing greater attention and awareness to PA, may be an important counterweight to this negativity bias, and in turn, may be mechanism reducing suicide risk.

**Positive Psychology Interventions (PPI).** Few PPIs have specifically been applied to patients with suicidal behavior. Two meta-analyses of PPI for depression and well-being found that these interventions have small to moderate effects in enhancing well-being and decreasing depressive symptoms and that effects are stronger for individual vs. group formats (28, 29). However, the vast majority of these interventions were with nonclinical populations, with nearly half of them conducted with college students. Only a handful of studies recruited from clinical or hospital settings (30-33). The few studies conducted with suicidal populations (not included in the meta-analyses) have yielded mixed findings. One recent study of 201 inpatient adults randomized to a 7-day program of either gratitude or food diary found significant between group differences on SI, psychological pain, hopelessness and optimism, favoring those in the gratitude condition (34). However, another study, in which 65 adult inpatients were randomized to a positive psychology exercise (e.g., gratitude letter) vs. a cognition-focused intervention (e.g., recalling daily events), found that contrary to the hypothesis, those randomized to the cognition-focused intervention had significantly greater improvement in depression, SI, optimism, and gratitude, compared to the positive psychology intervention (35). No PPIs for suicidal adolescents, to our knowledge, have been published with the exception of our pilot work.

**Rigor of the Prior Research.** As noted above, although a number of interventions for adolescent suicidal behavior have been tested, they focus on negative affect and the findings to date are modest. New approaches are indicated.

**Skills to Enhance Positivity (STEP).** We developed the STEP intervention for adolescents admitted to an inpatient psychiatric unit due to suicide risk. STEP is a multi-modal, adjunctive intervention involving 4 individual sessions delivered on the inpatient unit, followed by text or email messaging post-discharge to assess mood and “push” positive affect strategies for up to three months. While STEP borrows heavily from preceding positive psychology interventions, such as Seligman’s positive psychotherapy (PPT) (32), importantly, it is also distinct. STEP does not address happiness, optimism, or meaning, which may feel unattainable or invalidating to patients in acute distress. Rather, it focuses on increasing attention and awareness to positive emotions and experiences that may be easily discounted by the cognitive constriction that often accompanies SI and SB. Consequently, specific exercises designed to increase attentional awareness (i.e., psychoeducation on functions of emotions, mindfulness, gratitude, and savoring) are practiced, and, in this sense, STEP more closely aligns with acceptance-based approaches to treating depression, which have become increasingly common (36).

MPIs Yen and Spirito have conducted an open trial (N = 20) (37) and a pilot RCT (N = 52) (MH R34101272) (38) of STEP; both trials indicated excellent feasibility. The samples were characteristic of inpatient settings, i.e., high rates of comorbidity, especially mood disorders (92%), NSSI (96%), and past SA 52%). (See 43, 44 for details). We will recruit participants from the same site, thus anticipate the same clinical features in the proposed study. On average, 81% of in-person sessions were completed in the open trial, and 83% in the RCT. Participants responded to the text-messaging component of STEP on 74% of days in the open trial and 70% of days in the RCT. Based on feedback received from the open trial in which respondents requested text messaging be extended, we offered an optional 3-month extension of texts 3x/week to those randomized to STEP in the RCT; 52% opted for this extension without any financial incentives. Acceptability ratings for in-person sessions and text messaging, as well as the specific exercises introduced in STEP, were high for both adolescents and parents in both trials, with 95% in Open and 88% in RCT rating quality of services as either good or excellent.

In the RCT, over 6 months of f/u, 5 of 26 participants in the STEP condition (19%) vs. 10 of 26 in the Enhanced Treatment as Usual (ETAU; healthy habits texts) condition (38%) had a suicidal event. Six suicidal events were reported among STEP participants and 13 events were reported among ETAU participants. Thus, those randomized to STEP had 50% fewer events and 50% fewer participants engaging in suicidal events, compared to ETAU. This frequency count corresponds to a medium to large effect size *h* of .43. Furthermore, those in the STEP condition saw a more dramatic decrease in active SI from baseline compared to their worst week during the f/u period (STEP 49% vs. ETAU 19% decrease) (Fig 1). At baseline, 80% of STEP participants and 69% of ETAU participants reported active SI in the week preceding hospitalization. The percentages of those with active SI during their worst week of follow-up (f/u), dropped to 32% in STEP vs. 50% in ETAU (Cohen’s d = -0.74). Compared to ETAU, STEP had significantly greater improvement in depression (parent report on the Beck Depression Inventory)(39) at post-treatment (d=0.76; 95% CI 0.21-1.30) (38).

Our data also suggest that STEP engaged the target of PA. On the attentional dot probe task using words, groups did not differ at baseline or post treatment. However, a significant between group difference was found with those in the STEP condition showing more attention biased toward happy words at the 3-month f/u compared to the ETAU group, *t*(34) = 2.06, *p* = .048, η^2^ = .04. While our self-report assessments did not yield any significant between-group differences, stronger pre-post effects (from baseline to post-tx, and baseline to f/u) were observed in STEP on *gratitude* and *satisfaction with life*, with significant within group comparisons observed in STEP but not in ETAU for 3 out of 4 comparisons (effects sizes (Cohen’s *d*) ranging between 0.59-0.87). Furthermore, mood responses based on the six Modified Differential Emotions Scale (mDES)(25) items delivered daily to STEP participants, suggested that in 400 total days of full reporting (58% of surveyed days), 84% of STEP participants reported more days in which their PA > NA. We also observed a strong within-group effect on our secondary mechanism of negative affect for those in STEP (d = 1.01), although there was no statistically significant difference between groups. Finally, we have data supporting our hypothesized mechanisms are related to suicide outcomes. *Satisfaction with Life* at post-treatment predicted SE and active SI at the 6 month f/u, and these measures at the 3 month f/u, predicted SE at 6 month f/u. Adjusting for direction of effects, odds ratio (*OR)* ranged from 1.10 to 1.17; p = .04 to .003, respectively. Furthermore, PA (mDES) at 3 month f/u predicted 6 month active SI (adjusted OR = 2.33; p = .03), and NA (mDES) at post-treatment and 3 month f/u predicted 6 month SE and active SI (OR ranged from 2.69 to 4.94; p = .02 to .004).

These preliminary data importantly demonstrate the feasibility, and our capability, of mounting a large-scale trial and implementation study of the STEP intervention. We have the capacity to recruit adolescents, and to engage them in STEP. We also show evidence of efficacy and engagement with the target, although we recognize that the small sample size of the pilot trials leads to low precision of these effect size estimates.

**Significance.** As rates of suicide and suicidal behavior are on the rise in adolescents, our proposal has public health significance by targeting a high-risk, vulnerable, adolescent population during a time of acute need (Healthy People 2020 Objective MHMD HP 2020- 2: Reduce suicide attempts by adolescents). This application also strongly aligns with the NIMH 2020 Strategic Plan for Research, specifically objective 3.3. “test interventions for effectiveness in community practice settings” and 3.3.c “enhancing the practical relevance of effectiveness research via deployment-focused hybrid effectiveness-implementation studies,” as well as objective 8.4 of the National Strategy for Suicide Prevention to promote continuing care after hospital usage which in turn may reduce re-hospitalization, another significant cost containment and public health benefit. Our proposed adjunctive intervention is also clinically significant and innovative because it was developed to work synergistically with psychotherapies and designed such that it can be delivered either adjunctively or integrated as a module into another manualized protocol. The integration of remote reminders facilitates daily in-vivo practice of skills. We will be able to explore potential moderators that may inform which youth benefit from this treatment approach, with attention to biological sex and gender identity, severity of depressed mood, service level factors, (i.e., number of sessions received, engagement with the program) and fidelity of frontline staff to the delivery of STEP (40). Finally, **the rigor of the proposed research** is evident in the strong scientific premise as it is based on the research literature, including our own prospective naturalistic study, an open trial, and a pilot RCT. Furthermore, we gather data from multiple sources (participants, parents, staff), and propose multiple assessment modalities (clinical interviews, explicit self-report measures, implicit behavioral tasks and eye tracking) to assess primary and secondary outcomes, and utilize quantitative and qualitative methods to address our exploratory aim regarding implementation. Our conceptual model is premised on the Broaden and Build theory of positive affect – a model that has garnered abundant empirical support in social psychology laboratories but has yet to be translated towards efforts to prevent suicidal behavior. Our findings could build support for an ancillary mechanism to suicidality and further inform the positive valence domain of RDoC.

## Risk/Benefit Assessment

### Known Potential Risks

1. Potential coercion: It is possible that patients may feel coerced into participating.
   1. The risk of potential coercion will be minimized by following standard procedures for obtaining informed consent, which includes a statement that participation is completely voluntary. We will fully explain the study procedures, risks, benefits and alternatives to all prospective participants and their parent(s). Also, patients who do not consent or who withdraw at any time will receive usual clinical treatment with no prejudice.
2. Confidentiality and loss of privacy: We will be collecting considerable information about the participant and their parent that may create some distress and could cause social and psychological risk if released inappropriately. Additionally, participants in the intervention condition will be corresponding with the study by text messaging through their personal phone. Emails and phone calls may also be used to schedule interviews.
   1. All research personnel will receive training in research ethics. All information will be treated as confidential material and will be available only to research and clinical staff trained to deal appropriately with sensitive clinical issues. All PHI will be stored in REDCap and will require a log-in. We will identify participant research data by numeric ID only (i.e. no identifying information on research data) and maintaining any records containing potentially identifying information separate from any research data. Paper files will be stored in locked file cabinets; computer data files will be available only to authorized personnel and will be password protected. No names, only identification codes, will be used in presenting data in lectures, seminars, and papers. Transfer of any data from or to Brown University (and any of the participating sites: Beth Israel Deaconess Medical Center, Boston Children’s Hospital, Butler Hospital, Bradley Hospital) will be performed by trained research staff and will comply with all HIPAA guidelines including the removal of any personal health information. Participant confidentiality will be breeched only to protect the safety and welfare of research participants and only in accordance with state and federal law.
   2. We will take steps to mitigate the loss of confidentiality and privacy with emails, text messages, and phone calls, by reviewing with the participant, good practices to ensure privacy during the consent process. These include logging out of their email account after use, setting up a passcode to their phone, and not checking messages when other people are around. Research personnel are instructed to never leave voice messages with confidential information. You will have to enter a password to receive the text messages.
3. Risk of adverse events during the study: It is possible that some patients will have an adverse event during the study, including subsequent suicidal ideation or behavior or other adverse events.
   1. Since the intervention being evaluated in this study is an adjunctive intervention, participation in the study will not prohibit involvement in any other type of treatment. All subjects will be allowed (and/or encouraged) to participate in whatever type or amount of treatment is thought to be clinically indicated for the patient. Thus, participation in the study will not cause any subject to be deprived of a clinically appropriate treatment.
   2. Participants may be pulled out of regular group or milieu programming for study intervention sessions.
   3. All subjects in the study will receive enhanced monitoring of their clinical condition. We will obtain release of information forms and will communicate with the patient’s clinician(s) if clinical deterioration occurs. All patients will be monitored at each assessment point with respect to suicidality. Patients in ETAU will be monitored for suicidality based on a clinician’s standard practice. All patients in the experimental protocol will be monitored at every session of the intervention protocol with regard to suicidality.
   4. We will have procedures in place to respond to adverse events if reported. Please refer to 8.2.
   5. All adverse and serious adverse events will be recorded and reported, as outlined in the table below.

| **Table 1. Reportable event timetable** | | |
| --- | --- | --- |
| **Reportable Event** | **When is Event Reported to the IRB and NIMH DSMB** | **Reported By** |
| IRB/ISM/DSMB/OHRP/FDA Suspensions or Terminations | Any suspension or termination of approval must include a statement of the reason(s) for the action and must be reported promptly to the NIMH PO within **3 business days of receipt.** | Regulatory or Monitoring Entity and Investigator |
| Deaths related to study participation | Deaths must be reported immediately (no later than within **5 business days)** of the principal investigator first learning of the death. | Investigator |
| Unexpected [Serious Adverse Events](http://www.hhs.gov/ohrp/policy/advevntguid.html#Q6) related to study participation* | Reported within **5 business days** of the study team becoming aware of the SAE | Investigator |
| [Unanticipated Problems Involving Risks to Subjects or Others](http://www.hhs.gov/ohrp/policy/advevntguid.html#Q1) | Reported to the NIMH and IRB within 5 **business days** of the investigator learning of the event. | Investigator |
| [Adverse Event](http://www.hhs.gov/ohrp/policy/advevntguid.html#AA) | \|  \| For all AEs and SAEs that are deemed expected and/or unrelated to the study, a summary should be submitted with each tri-annual DSMB report for SAEs, and annual progress report for AEs to the DSMB. The IRB will receive all of these reports annually. \| \| --- \| --- \| | Investigator |
| Protocol Deviations  Protocol Violations | \|  \| With the annual IRB progress report. \| \| --- \| --- \|   Annual IRB progress report and 3x per year to the DSMB. | Investigator |

Note: although NIH recommends 10 days, local IRB requests 5 days

### Known Potential Benefits

All individuals participating in this research will benefit by having a thorough baseline psychological assessment as well as potentially effective intervention which may result in a decrease in suicidal risk. The potential benefits to individuals outweigh potential risks, particularly in offering them the chance to better understand the nature of their suicidal behavior and to receive a baseline psychological evaluation. On a larger scale, the information obtained from this study will further our understanding of how to treat adolescents who attempt suicide.

### Assessment of Potential Risks and Benefits

Risk associated with participation in the study is low. Study participation will provide patients with additional clinical care and assessment without additional cost. Research on preventing future suicide attempts in high-risk groups is greatly needed due to the prevalence of the problem and the lack of extensive research in this area. Therefore, the benefits are anticipated to exceed the risks for participants.

# OBJECTIVES AND ENDPOINTS

| OBJECTIVES | ENDPOINTS | JUSTIFICATION FOR ENDPOINTS | PUTATIVE MECHANISMS OF ACTION |
| --- | --- | --- | --- |
| Primary |  |  |  |
| To examine the effectiveness of STEP in reducing suicidal events (suicide attempt or emergency intervention to intercede attempt, i.e. ED evaluation or psychiatric hospitalization), active suicidal ideation (with intent or plan), and depression, compared to ETAU. | Suicidal events and active suicidal ideation as assessed by the Columbia-Suicide Severity Rating Scale (C-SSRS) interview, and depression as assessed by the Beck Depression Inventory (BDI-II) at 6 month follow-up (primary endpoint) and suicidal events at 12 month follow-up (secondary endpoint) | Six-month follow-up is selected as the primary end-point time as numerous studies have shown this to be a particularly high-risk time for repeat suicidal behaviors. Twelve months allow us to assess the long-term effectiveness. | NA |
| Secondary |  |  |  |
| To examine engagement of the hypothesized mechanisms of positive affect (PA) and negative affect (NA). | Attention to PA and NA as assessed by implicit cognitive tasks and self-report forms (See 1.3) at 3 month follow-up (primary) and 6 month follow-up (secondary) endpoint. | PA and NA are hypothesized to be causal mechanisms. Thus these assessments will see if we engaged our treatment target of PA and NA. | PA and NA are hypothesized to be causal mechanisms. |
| Tertiary/Exploratory |  |  |  |
| To examine whether hypothesized mechanisms mediate reduction of suicidal events and ideation. | Attention to PA and NA as assessed by implicit cognitive tasks and self-report forms (See 1.3) at 3 month follow-up (primary) and 6 month follow-up (secondary) endpoint. | The PA and NA assessments, using both implicit and subjective assessments, will be examined as mediators. Specifically, PA and NA at 3 month f/u is hypothesized to mediate the relationship between study intervention and clinical outcomes at the 6 month f/u; and PA and NA at 6 month f/u is hypothesized to mediate the relationship between study intervention and clinical outcomes at the 12 month f/u. | PA and NA will be examined as mediators. |

# STUDY DESIGN

## Overall Design

This is a randomized controlled trial (RCT) of Skills to Enhance Positivity (STEP) vs. Enhanced Treatment as Usual (ETAU). It is hypothesized that those randomized to STEP, compared to ETAU, will have lower rates of suicide events, active SI, and depression over the 6-month follow-up period (primary endpoint), and over 12 months of follow-up (secondary endpoint). Recruitment will occur at three sites, Boston Children’s Hospital, Butler Hospital, and Bradley Hospital. Eligible individuals who consent to participate will be randomly assigned to either STEP or ETAU in a 1:1 allocation after the baseline assessment. Co-I Jones will prepare a stratified [by study site, history of SB (yes/no), and sexual or gender minority (yes/no)} randomization schedule using random blocks and provide this to our REDCap administrator; study staff will not have access to randomization tables). Research staff will be able to obtain assignments in real time once consent and stratification variable information are recorded. We use random block sizes to prevent guessing of pending treatment assignments.

The study intervention, Skills to Enhance Positivity (STEP), is an adjunctive psychosocial intervention that involves 4 in-person sessions during a participant’s inpatient admission, followed by three months of text or email messaging for mood monitoring and skills delivery. (If participant has a shorter length of stay or is discharging early, the STEP sessions may be shortened so that the intervention can be delivered prior to discharge). The sessions and skills delivered via the text or email messages focus on the functions of positive affect and ways to bring greater attention to and awareness of positive emotions and experiences on a daily basis. Four videos with content derived directly from the treatment manual will be used to support the delivery of the STEP skills that are covered in the in-person sessions.

Parents/guardians will be provided with a handout containing information about the study, contact information, and details on how they can best support their child throughout the duration of the study. The comparison condition (ETAU) consists of treatment as usual on the unit which is enhanced by discharge plan which includes reminders of their safety plan and that key phone numbers (supports, suicide hotlines) are preprogrammed for easy access. Participants will receive the messages on a daily basis for 30 days with reminders that their safety plan and phone numbers are on their phone. ETAU participants will also receive reminders to practice some Healthy Habits and reminders to look at their safety plan. Similar to the STEP condition and as a means to control for contact time, after 30 days, ETAU participants will be placed on a tapered schedule of receiving alerts 3 times per week for an additional 60 days.

**Primary Aims are described above under Section 3, Objectives and Endpoints. There is an additional secondary aim which reads as follows:**

*Examine elements supporting external validity*. Acceptability, appropriateness, and feasibility of STEP to patients, parents, clinicians, and inpatient unit administrators will be assessed by: 1) a survey consisting of brief, standardized measures of these items, and 2) qualitative interviews to further explore these perceptions. This will be assessed pre-implementation of the RCT to enhance the feasibility of our trial (details of the pre-implementation phase are not discussed in this document because they precede the RCT), and post-RCT implementation to ascertain facilitators and barriers. These findings will be used to: 1) modify STEP to address potential barriers, and 2) develop implementation strategies designed to overcome these challenges for testing in a future Hybrid Type III effectiveness-implementation trial. The measures used in post-RCT implementation are listed above in the measures chart. Providers, adolescents and guardians will participate in qualitative interviews and complete self-report measures (about intervention acceptability, appropriateness, feasibility, as outlined at baseline and varying schedules at the 3, 6, and 12-month follow-up assessments). Providers only will complete additional measures at baseline, 6, and 12-month follow-ups. These self-report measures will examine evidence-based practice attitudes, implementation complete, and implementation readiness.

## Scientific Rationale for Study Design

The study design is an RCT comparing the study intervention to an enhanced treatment as usual condition. All participants receive equivalent inpatient care with the exception of receiving either STEP sessions or. TAU therapy sessions, depending on the condition to which they are designed. STEP participants will be pulled from regular therapeutic programming for their 4 STEP sessions (while ETAU will be in therapy sessions). After discharge, ETAU get prompts to remind them of their safety plan or practice a Healthy Habit with the same frequency as STEP participants. Both conditions receive the exact same assessments and the passage of time is equivalent. Therefore, we do not anticipate any problems associated with the control group in light of participants’ risk for suicidal behavior, as they will continue to receive ongoing treatment on the inpatient unit and are able to engage in outpatient treatment after discharge.

## Justification for Intervention

STEP involves four in-person sessions on the inpatient unit, as well as remote delivery via text or email for three months post-discharge. Testing a protocol that is delivered during inpatient hospitalization maximizes the use of frontline milieu staff to deliver a low-cost intervention. The use of multiple modes of delivery, i.e., an in-person delivery phase, to occur on the inpatient unit, combined with a remote delivery phase, to occur upon discharge to their home environment, facilitates engagement with high-risk youth who may encounter barriers to subsequent care, through low-cost, broadly accessible means. The number of sessions and duration of intervention is informed by feedback from a prior pilot trial of this intervention. The minimum-acceptable participation will be defined as one in-person session and any engagement with the text/email messaging program. To accommodate participants who might have a shortened length of stay in the hospital, the STEP sessions might be compressed at times so that teens have the complete intervention delivered prior to discharge. If teens have their phones taken away, parents will be asked to allow their child to access the text messages from the STEP protocol when they are received at a pre-specified time (at the same time each day) for a time-limited duration, for the purposes of the study protocol. Alternatively, if it is not possible for the teen to have access to their phone, participants will instead receive the messages through email and REDCap.

## End-of-Study Definition

A participant is considered to have completed the study if he or she has completed the baseline assessment, and the 6-month and 12-month follow-up assessments.

The end of the study is defined as completion of the 12-month follow-up assessment shown in the Schedule of Activities (SoA), **Section 1.3**.

# STUDY POPULATION

The primary study population is adolescents psychiatrically hospitalized due to suicidal ideation or behavior. Parents will also be enrolled as study participants because they are asked to participate in study assessments, they will solely be informants of their child’s symptoms and experiences. All recruitment sites (Butler Hospital, Bradley Hospital, and Boston Children’s Hospital) have a broad payor mix and include Medicaid and uninsured patients. These participants (and their guardians) are described below.

In addition, at the end of the trial, acceptability, appropriateness, and feasibility of STEP to clinicians, and administrators will be assessed by: 1) a survey consisting of brief, standardized measures of acceptability, appropriateness, and feasibility, and 2) qualitative interviews to further explore these perceptions. These findings will be used to: 1) modify STEP to address potential barriers, and 2) develop implementation strategies designed to overcome these challenges for testing in a future Hybrid Type III effectiveness-implementation trial.

## Inclusion Criteria

In order to be eligible to participate in this study, an individual must meet all of the following criteria:

1. Provision of signed and dated informed consent form
2. For children, informed assent and parental informed consent to participate in the study
3. Age 12-18 years
4. Hospitalized on an inpatient psychiatric unit due to suicide risk (attempt, ideation, threat) as noted in the Admission note
5. Past-month attempts or ideation as verified by the Columbia Suicide Severity Rating Scale interview
6. Willingness to adhere to the STEP regimen
7. Participants must have access to their own phone.
8. Participants must be proficient in English, and a parent fluent in either English or Spanish

## Exclusion Criteria

An individual who meets any of the following criteria will be excluded from participation in this study:

1. Acute psychotic disorders or cognitive deficits that would preclude full understanding of the protocol, intervention, and assessment materials. Determination of mental status as it relates to patients’ ability to fully participate in this study will be based on chart review and consultation with unit psychiatrists. Similar criteria will be used if cognitive deficits or psychosis symptom occur at follow-up appointments. The research team will assess whether these deficits would preclude full understanding of the assessment protocol. If so, they will be withdrawn. We anticipate that most participants will remain in the study because fleeting cognitive symptoms may occur in disorders other than psychosis spectrum disorders.
2. Patients who are admitted from, or have a planned discharge to, a residential treatment facility will be ineligible. However, those admitted to residential treatment *after* enrollment in our study will remain in the study.

## Screen Failures

Screen failures are defined as participants who consent to participate in this study, but are not subsequently assigned to the study intervention or entered in the study because they did not meet inclusion criteria upon baseline assessment. Individuals who do not meet the criteria for participation in this trial (screen failure) because of meeting one or more exclusion criteria will not be rescreened.

## Strategies for Recruitment and Retention

**1. Recruitment Procedure and Referral sources**.

Participants will be recruited from the adolescent inpatient units of Butler Hospital and Bradley Hospital in Providence, RI and Boston Children’s Hospital (BCH) in Boston, MA. Participants will be recruited shortly after hospital admission. Research staff, who are authorized to access patient’s admission information, will approach eligible prospective participants and will provide information about the study to parents/guardians including an oral and written description of study procedures, appointments, compensation for participation, potential benefits and risks, and confidentiality. Families who provide parental consent and adolescent assent will complete the intake battery as soon as possible.

We target recruiting 108 participants at each clinical site (BCH in Boston and the combined Butler/Bradley site in Providence) over 2 years. Excluding summer months when census is low, we plan to recruit six participants per month from each unit. The adolescent inpatient psychiatric unit at Butler Hospital has a 15-bed capacity and has over 400 unique admissions each year, with approximately 75% being admitted due to suicide risk. The adolescent inpatient psychiatric unit at BCH has 16 beds and over 325 unique admissions per year. Thus, our recruitment plan is very feasible. Furthermore, Dr. Spirito’s most recent RCT with adolescent inpatients met its recruitment goal (Esposito-Smythers et al., 2019) as did the pilot RCT (R34) of the STEP intervention (Yen et al., 2019). In the R34, 78% of those eligible per chart screening were approached about the study. Of those approached 68% were enrolled into STEP.

**2. Procedures that will be used to monitor enrollment and track/retain participants for follow-up assessments**.

Enrollment will be monitored and discussed in weekly conjoint meetings with the PIs at each site via Zoom. These meetings will be used to discuss ways to improve enrollment and complete follow-up contacts with participants. Drs. Yen and Spirito have used these procedures with success in past projects. Once enrolled in the study, we will take steps to ensure that we retain families in the follow-up portion of the study. A tracking database using SQL will be developed specifically for this project and will include all due dates for each participant and to-do items for each RA, by a professional data management team. Adolescents and parents will be asked for their preferred contact mode (text, phone, and/or email) and will be contacted prior to their 3, 6 and 12-month follow-up appointments. In the R34 based at Butler Hospital, a very high retention rate was maintained, with 80% of participants completing the final follow-up assessment. Co-I O’Brien has collaborated with both PIs on an adjunctive app-based intervention (R34 AA025763) delivered on the inpatient unit at BCH. She has collaborated with the inpatient team at BCH on multiple studies with suicidal adolescents over the past six years and enrolled 186 total subjects across four studies. She has an 84% retention rate for her studies at their 3-month follow-up appointments. Thus, previous research at this site employing similar procedures supports the feasibility of the current study design.

Drawing on our experiences conducting longitudinal research with adolescents, our retention strategies will include frequent phone, text message, and email contact. We will collect multiple forms of contact (e.g., email address, cell phone, and home phone). We will also ask families to complete locator forms (i.e., contact information for a family friend or relative) to be used in the event that their contact information changes and we have difficulty reaching them. We have found these strategies helpful for retaining and reconnecting with families in our studies. Whenever possible, the same interviewer will initiate all contact, including reminder contacts, and facilitate the appointments with any given participant. We have found that this strategy is helpful for maintaining rapport and participant retention. We will schedule participants by phone, text, or email two weeks in advance of the target date, and send reminders the day prior to the appointment. We will also employ motivational interviewing techniques at the baseline assessment to maximize participant sense of agency and engagement in the study. Specifically, at the end of their baseline assessment, we will elicit from adolescents and their parents the benefits and importance they perceive of the study (e.g., personal benefits or benefits to the larger community), as well as potential barriers to adherence to follow-up participation. We will then collaborate with participants to devise an action plan for addressing these potential barriers.

Additionally, arrangements for home visits, appointments at community centers close to participants’ homes, or cab vouchers will be made with families unable to participate in the follow-up assessment because of lack of transportation. Alternatively, we can conduct the majority of the assessment virtually, but arrange to do the behavioral task in-person. Finally, our consent form will include permission for the duration of the study to audit electronic medical records at Boston Children’s Hospital, Butler Hospital as well as Bradley Hospital, the only other hospital with an adolescent inpatient psychiatric unit in RI and the surrounding area. Audits of medical records will be used to obtain new contact information as needed, as well as to collect data on ED and hospital inpatient admissions.

**3. Strategies that will be used to ensure a diverse, representative sample**.

In our R34 pilot study of STEP, 68% of our sample reported being female and 32% reported being male. When non-binary options are provided, approximately 5% identified as transgender or non-binary. Furthermore, 52% of our sample identified as a sexual or gender minority (LGBTQ).

Based on census statistics from both Butler Hospital and Boston Children’s Hospital, as well as our past recruitment efforts, we expect approximately 20% of our sample to identify as Hispanic/Latinx. With respect to race, we project that the sample will consist of 77% Caucasian, 5% African-American, and 13% multi-racial participants.

To ensure a diverse representative sample, in the case of multiple possible participants available for enrollment, those of minority status who meet eligibility criteria will be given priority in order to increase the percentage of minorities in the study sample.

**4. Potential recruitment/enrollment challenges and strategies that can be implemented in the event of enrollment shortfalls**.

Given the success of the R34 in recruiting patients, and the interest in both sites in conducting an adjunctive intervention for hospitalized adolescents, we do not anticipate any problems in enrollment. We believe this study could be completed at the site of the R34, Butler Hospital, but decided on a two-site study for this application to both increase generalizability and expediency, and to ensure we could meet our recruitment goals. Staff buy-in is key to successful recruitment and it is very strong in this application. In addition, we release our clinical findings, with the families’ permission, to the clinical team for treatment planning purposes, a significant benefit of participating in the research. In our experience, the clinical treatment team has found inpatients’ participation to be beneficial because of the additional clinical material that it provides, and therefore they encourage parents to participate in the research. In the event that we are below our recruitment goals, we can plan to recruit from Bradley Hospital in Providence where Dr. Spirito has recruited adolescents in other studies, and the CBAT unit of Boston Children’s Hospital.

**5. Evidence to support the feasibility of enrollment, including descriptions of prior experiences and yield from research efforts employing similar referral sources and/or strategies**.

In a recent R01 treatment study, Dr. Spirito reached his target and enrolled 85% of eligible psychiatrically hospitalized patients (Esposito-Smythers et al, 2019). Families typically appreciate receiving a more in-depth evaluation and the opportunity to receive treatment in a research study.

Dr. Yen has successfully recruited for 4 studies from the Butler adolescent inpatient unit. Most importantly, her R34 that was the pilot for this R01 successfully met its recruitment goals. In her most recent study which recruited from Butler Hospital, she has surpassed her recruitment goals in recruiting sexual and gender minority youth at risk for suicidal behaviors.

Dr. O’Brien has successfully recruited suicidal adolescents as a PI for four different studies from the site proposed in this application, with average retention rates of approximately 85%, demonstrating her capability to enroll and retain adolescents for the proposed study. Dr. Spirito who has been a Co-I on projects at Butler Hospital with PI Yen and at BCH with site PI O’Brien, also has had high retention rates following adolescents from inpatient units.

**6. Participant Incentives**.

Participants and parents will be administered baseline assessment and the 3-, 6-, and 12-month follow-up separately. Each participant and parent will be compensated $50 for the baseline assessment, and adolescent participants will receive a $20 bonus for completing the computer task. For the 3- and 6-month assessments, the participant and the parent will each receive $50, with a $20 incentive for on-time assessments that are completed within two weeks of the follow-up due date. The adolescent also has an opportunity to earn a $20 bonus for completing the computer task. Each participant and parent will be compensated $30 for the 12-month assessment (with a $20 incentive for on-time assessments that are completed within two weeks of the follow-up due date).

Compensation will be in the form of cash, money order, or ClinCard (a refillable payment card). This amount is commensurate with the time spent for doing assessments and is not excessive or coercive. Compensation will be paid directly to the participant for their participation and directly to the parent if they have participated as an informant. Stakeholders are compensated $50.

# STUDY INTERVENTION(S) OR EXPERIMENTAL MANIPULATION(S)

## Study Intervention(s) or Experimental Manipulation(s) Administration

### Study Intervention or Experimental Manipulation Description

We developed the STEP intervention (MH R34101272) for adolescents admitted to an inpatient psychiatric unit due to suicide risk. STEP is a multi-modal, adjunctive intervention involving 4 individual sessions delivered on the inpatient unit, followed by text or email delivered messages to assess mood and “push” positive affect strategies for up to three months post-discharge. While STEP borrows heavily from preceding positive psychology interventions, such as Seligman’s positive psychotherapy (PPT) (32), importantly, it is also distinct. STEP does not address happiness, optimism, or meaning, which may feel unattainable or invalidating to patients in acute distress. Rather, it focuses on increasing attention and awareness to positive emotions and experiences that may be easily discounted by the cognitive constriction that often accompanies SI and SB. STEP is based on the Broaden and Build theory of positive affect. Consequently, specific exercises designed to increase attentional awareness (i.e., psychoeducation on functions of emotions, mindfulness, gratitude, and savoring) are practiced, and, in this sense, STEP more closely aligns with acceptance-based approaches to treating depression, which have become increasingly common (36).

### Administration and/or Dosing

*In-person phase*. STEP consists of 4 individual, treatment sessions typically completed in days 4 – 7 on the unit. In the pilot trial, all participants were started on the inpatient unit and 65% completed all sessions on the unit with the remainder, typically session 4, scheduled after discharge. In the current study, we will add procedures to increase parent engagement with STEP via a handout which will be provided after consenting and randomization to the STEP condition at the time of discharge. This handout describes the study goals and outlines ways in which parents/guardians can support their children throughout the study. The content of the sessions is described in the table below. Participants will be pulled from other therapeutic activities on the unit for their STEP session so that STEP participants will not receive more therapeutic contacts while hospitalized than the ETAU participants. Sessions are designed to maximize flexibility such that if not all of the content is delivered in one session, it can be delivered in the following session. For participants who have a shorter length of stay or are discharged early, the individual STEP sessions might be compressed to be able to deliver the intervention in a timely manner prior to their discharge. Four videos with content derived from the treatment manual will be used to help supplement the skills covered in the STEP sessions.

| Table 2. STEP In-Person Sessions |  |
| --- | --- |
| **Session 1: Rapport, Rationale and Psychoeducation**   - Brief explanation of program and rationale - Context for admission? - Reasons for living - Protective factors, strengths and resources - Introduce adaptive functions of both PA and NA - Discussion and examples of negativity bias - Introduce simplified version of Broaden and Build theory - Introduce concept of positivity ratio, bank account - Explain objective of daily mood monitoring - Discuss what STEP is not (e.g., toxic positivity, individual therapy) - Attempt to establish rapport | **Session 2: Mindfulness / Meditation**   - Review rationale of increasing attention to positive affect and experiences - What is Mindfulness? What does it do? - Ask about past experiences – likes / dislikes - Discuss that there are different ways of practicing mindfulness - Introduce Mindfulness Meditation grounded in observing breath - Practice a mindfulness exercise (10 deep breaths or other) - Introduce Movement Meditation - Practice a movement meditation (PMR, walking, yoga stretch) - Introduce Compassion/Mantra Meditation - Practice mantra meditation (identify a mantra) - Personalized selection of skills, evaluate feasibility of each type of practice (create buy-in) - Encourage independent practice - Identify barriers to practice in home environment - Troubleshoot |
| **Session 3: Gratitude**   - Review rationale of increasing attention to positive affect and experiences - Review of exercises from previous session including problems or barriers - Why do Gratitude exercises? - Ask about past experiences – likes / dislikes - Different ways of practice - Introduce 3 Good Things - Practice 3 Good Things - Introduce Expression of Gratitude - Envision Expression of Gratitude - Introduce Acts of Kindness – offer examples and discuss impact - Personalized selection of skills, evaluate feasibility of each type of practice (create buy-in) - Encourage independent practice - Identify barriers to practice in home environment | **Session 4: Savoring**   - Review rationale of increasing attention to positive affect and experiences - Review of exercises from previous session including problems or barriers - Why do Savoring exercises? - Ask about past experiences – likes / dislikes - Different ways of practice - Introduce Sharing Good Things - Practice Sharing Good Things - Introduce Simple Savoring - Practice Simple Savoring - Introduce Journaling of Positive Events – discuss feasibility - Personalized selection of skills, evaluate feasibility of each type of practice (create buy-in) - Encourage independent practice - Troubleshoot - Review expectations for remote delivery phase |

***Remote delivery phase.*** The remote delivery phase of STEP begins immediately after discharge. All participants will be asked to take a photo of their safety plan and to have phone numbers of supportive resources stored into their contacts. Those randomized to STEP will be sent an automated sequence of text messages, starting with a password entry as an additional confidentiality safeguard. They are then asked to complete a set of 6 mood monitoring questions from the mDES (e.g., “*How glad, happy, joyful, do you feel RIGHT NOW? Enter any # from 1 to 5 (1 = not at all, 5 = extremely*)”), followed by a request for the participant to select the type of exercise (i.e., mindfulness meditation, gratitude, or savoring) that they would like to practice *(“Choose the type of message you would like to receive right now: 1 = mindfulness; 2 = gratitude; 3 = savoring”)*. In response, the participant will receive a corresponding exercise to practice (e.g., “*share something positive with a friend or family member*”). Participants will also be asked if they had practiced a positive affect exercise the preceding day. If the participant does not respond after 2 alerts, a random exercise practice reminder will be automatically sent from a bank of exercise reminders. Therefore, participants will always receive a reminder to practice a positive affect exercise regardless of whether or not they engaged with the program. These procedures are the same as the pilot RCT, in which we have demonstrated feasibility of the approach. No questions about SI will be asked; nonetheless, a message will be displayed to let users know that no one is reading their responses and that if they are experiencing a mental health crisis, to contact their provider and/or Emergency Department. After the 30 days of daily practice reminders, participants will be placed on a tapered schedule of three times per week for an additional 60 days. After that time, they will no longer receive alerts.

The comparison condition consists of treatment as usual on the unit which is enhanced by asking participants to take a photo of their safety plan to store on their phone, and to ask participants to store phone numbers of supportive contacts on their phone. Participants will receive texts or emails on a daily basis for 30 days with reminders to check their safety plan and other healthy habit reminders. This procedure helps control for contact time by equating the two conditions. Unlike the STEP condition they will not receive the mood monitoring questions or positive affect skills as these are conceptualized as part of the study intervention. ETAU participants will be placed on a tapered schedule of receiving alerts 3 times per week for an additional 60 days. After 3 months, they will no longer receive alerts.

## Fidelity

### Interventionist Training and Tracking

**Therapist Training**. Two or more counselors (at least two on each clinical unit) will be trained to administer STEP. The unit chiefs on each unit will select staff, e.g. unit staff, mental health counselors, or social workers, that they believe should conduct the intervention and then offer the opportunity to receive training to the selected group of staff members. Staff who volunteer will be assured that any study-related performance evaluations performed by the research team will not be shared with their clinical supervisor. Training will follow the same protocol as in the pilot study. It will draw primarily from the STEP manual and will include written materials as well as didactic discussion of the goals of STEP. The training will also include reviewing tapes of selected sessions conducted by Dr. Yen from the pilot development study. Role plays will be utilized to demonstrate how exercises should be taught and how to personalize STEP. After completion of the didactic training, each provider will participate in mock sessions for 3 cases. Based on our experiences, we will develop an online accessible training protocol, to be used for future trainings. To ensure treatment fidelity, we will provide weekly supervision, review audiotaped role plays, utilize self-rated adherence checklists, and provide immediate feedback to the therapists. Periodic booster trainings will be conducted as needed.

**Treatment Integrity.** Dr. Yen will be the primary supervisor for STEP and will provide ongoing supervision, along with Drs. O’Brien, Spirito, and Wolff. To ensure treatment integrity, as part of the initial training, clinicians will be asked to administer STEP in a series of role plays, which will be rated by both the clinician-in-training and one of the study investigators. *Role plays will occur as part of the initial training, and again after the first 5 participants have been completed by the clinician.* Adherence and competency will be assessed using a checklist and competency rating scale developed by MPI Yen during the treatment development study. The scale included 39 items covering material from all 4 sessions (approximately 10 per session that reflect targets for that session). Each item is rated on adherence (dichotomous rating) and competency (along a 7-point Likert scale ranging from 0 to 6, poor to excellent). Adherence over 80% and competency ratings with a mean above 3.0 (satisfactory) will be considered acceptable. Furthermore, concordance of self-rated and investigator-rated adherence should reach adequate reliability defined as kappa>.70. After these benchmarks, the clinician will be asked to rate 20% of their sessions on adherence and competency. Self-ratings will both serve as a good reminder of the key components of the intervention, and also provide a metric of treatment fidelity.

## Measures to Minimize Bias: Randomization and Blinding

Eligible individuals who consent to participate will be randomly assigned to either STEP or ETAU. Co-I Jones will prepare a stratified (by study site, history of SB, and sexual or gender minority) randomization schedule using random blocks and provide this to our REDCap administrator; study staff will not have access to randomization tables). Research staff will be able to obtain assignments in real time once consent and stratification variable information are recorded. We use random block sizes to prevent guessing of pending treatment assignments. We stratify on history of past SB, because this factor has been shown to be a very strong predictor of a repeat hospitalization (6) and sexual or gender minority because this group is five times more likely to make a suicide attempt compared to heterosexual youth (51).

The assessment battery will be administered at baseline (prior to randomization) by the clinical interventionist. At the end of the active phase (3 months post-baseline), and at the 6- and 12-month follow-up intervals, assessments will be conducted by a blind evaluator, a research assistant located at a different site. For example, if the baseline assessment and sessions are conducted by the clinical staff at Butler Hospital or Bradley Hospital, the f/u assessments will be conducted by the Brown-based RA in another nearby building. Similarly, if baseline assessments and sessions are conducted by the clinical staff at BCH, the f/u assessments will be conducted by a Beth Israel Deaconess Medical Center (BIDMC) RA in another nearby building. The physical proximity of our sites (Brown to Butler/Bradley, and BIDMC to BCH) makes our plan to ensure blindness feasible.

In the event of inadvertent unblinding, the follow-up assessments will be conducted by another Research Assistant.

*6.4.* Study Intervention/Experimental Manipulation Adherence

Study intervention adherence will be tracked by the clinician via a clinician dashboard in REDCap. There will be tracking of attendance for in-person sessions, as well as tracking on whether there were any compressed sessions. Remote delivery adherence will be tracked based on the number of times the participant engages with the text messaging program, completion of the mood monitoring questions, and their response to the question of whether they have practiced the positive affect skill from the previous day.

## Concomitant Therapy

For this protocol, participants engage in other treatments and there are no restrictions on medications. Psychotropic medication usage will be assessed at each assessment time point and documented in the relevant Medication Compliance Questionnaire, and any psychosocial interventions will be tracked using the Treatment History Interview.

### Rescue Therapy

In the event of significant concern of suicide risk, study clinicians or investigators may suggest to the parent of a study participant that they bring their child to be evaluated in the emergency department. A detailed safety protocol is attached as an appendix, and described in Section 8.2.

# STUDY INTERVENTION/EXPERIMENTAL MANIPULATION DISCONTINUATION AND PARTICIPANT DISCONTINUATION/WITHDRAWAL

## Discontinuation of Study Intervention/Experimental Manipulation

When a subject discontinues from the study intervention, whether voluntarily or involuntarily, but not from the study, remaining study procedures will be completed as indicated by the study protocol. If a clinically significant finding is identified (including, but not limited to changes from baseline) after enrollment, the investigator or qualified designee will determine if any change in participant management is needed. Any new clinically relevant finding will be reported as an adverse event (AE).

The data to be collected at the time of study intervention discontinuation will include the following:

- The reason(s) for discontinuing the participant from the intervention, and methods for determining the need to discontinue
- If the participant is due to complete assessments within 2 weeks of being discontinued from the study intervention, those assessments will be administered at the time of discontinuation; if the next scheduled assessments are more than 2 weeks from the discontinuation date, the discontinued participant will wait for the next scheduled assessment. Thereafter, the participant will be included in all future scheduled assessments, even though not participating in the intervention.

A participant may be involuntarily discontinued from the study intervention if the participant has an unexpected adverse reaction to intervention content, develops psychiatric symptoms that make continuation of the intervention impossible, or if clinical staff deem it is not clinically appropriate to continue the intervention during the psychiatric admission. The participant will also no longer receive the messages, if they are discontinued from the study.

## Participant Discontinuation/Withdrawal from the Study

Participants are free to withdraw from participation in the study at any time upon request.

An investigator may discontinue a participant from the study for the following reasons:

- Significant study intervention non-compliance, i.e. with respect to the individual sessions, unless varying compliance is an aspect of the study objectives
- Lost-to-follow up; unable to contact subject (see **Section 7.3, Lost to Follow-Up**)
- Any event or medical condition or situation occurs such that continued collection of follow-up study data would not be in the best interest of the participant or might require an additional treatment that would confound the interpretation of the study
- The participant meets an exclusion criterion (either newly developed or not previously recognized) that precludes further study participation

The reason for participant discontinuation or withdrawal from the study will be recorded on the Case Report Form (CRF). Subjects who sign the informed consent form and are randomized but do not receive the study intervention may be replaced. Subjects who sign the informed consent form, and are randomized and receive the study intervention, and subsequently withdraw, or are discontinued from the study, will not be replaced.

## Lost to Follow-Up

A participant will be considered lost to follow-up if he or she fails to return for 3 scheduled follow-up assessment visits and study staff are unable to contact the participant after at least 3 attempts.

The following actions must be taken if a participant fails to return to the clinic for a follow up assessment visit:

- The site will attempt to contact the participant, reschedule the missed visit within two weeks, counsel the participant on the importance of maintaining the assigned visit schedule and ascertain if the participant wishes to and/or should continue in the study
- Before a participant is deemed lost to follow-up, the investigator or designee will make every effort to regain contact with the participant (where possible, 3 telephone calls and, if necessary, a certified letter to the participant’s last known mailing address or local equivalent methods). These contact attempts will be documented in the participant’s study file.
- Should the participant continue to be unreachable, he or she will be considered to have withdrawn from the study with a primary reason of lost to follow-up.

# STUDY ASSESSMENTS AND PROCEDURES

## Endpoint and Other Non-Safety Assessments

Please refer to Section 1.3, Schedule of Activities to see list of assessments, assessment modality, and timetable of specific administrations. Of note, guardians will be mainly reporting on adolescent thoughts, feelings, and behaviors for the assessment areas described below.

Demographics and Background Variables. Routine demographic variables will be collected. We will ask questions from the overview of the *Kiddie Schedule for Affective Disorders and Schizophrenia – Present and Lifetime version (KSADS-PL*)(52) to assess for current living situation, legal involvement, history of abuse, family psychiatric history, treatment history and payor information. Self-report forms will be used to ask about sexual and gender identity.

Suicidal Events. Suicidal events (operationalized as a composite score of suicide attempts and inpatient hospital/ED admissions)(42) will be assessed with the *Columbia-Suicide Severity Rating Scale* (50) and the *Child and Adolescent Services Assessment* (53). The C-SSRS assesses the range of suicidal behavior including preparatory acts, aborted attempt and interrupted attempt, and passive and active SI, as well as intensity of ideation. At f/u, suicidality since the last assessment and in the prior week will be assessed. The CASA is a semi-structured interview that obtains information about service use across multiple sectors (e.g., juvenile justices, schools). Adequate reliability has been demonstrated for this measure (ICCs=.74-.76)(53). Interviews will be administered at baseline, 3-, 6-, and 12-months f/u, with the 6-month follow-up as our primary outcome (Ho1A). The CASA will be used to systematically assess ED evaluations and psychiatric hospitalizations.

Suicidal Ideation. Active SI will also be assessed using the C-SSRS; specifically, we will assess active SI, operationalized by a score of 3 or higher on the suicide ideation questions of C-SSRS, using their highest level of ideation during the worst week of the f/u interval. We will also obtain weekly ratings of SI using the methodology of the *Longitudinal Interval Follow-Up Evaluation* (LIFE)(54), at the 3-, 6-, and 12 month f/u’s. The LIFE format asks participants to rate their level of SI in the preceding interval on a 6-point psychiatric status rating (PSR) scale. This methodology, which yields weekly scores, as applied to the assessment of SI in adolescents has been successfully implemented in at least three NIMH-funded studies for which MPI Yen was a PI (K23MH069904, MH090137, MH059929). Our data from adolescents admitted to the Butler adolescent unit and followed for 6 months after discharge (K23MH069904) demonstrate a good range of variability in SI PSR scores (e.g., 20% clinically significant SI; 36% no weeks clinically significant SI, 6% chronic, unremitting, high levels of SI for all weeks of f/u). We will also administer the Suicide Ideation Questionnaire, a 30-item self-report instrument with good psychometric properties in adolescents.

Diagnostic / Symptom Rating Scales. Depression will be assessed by the *Beck Depression Inventory (BDI-II)* (39) administered to both adolescent and parent (about teen). The BDI-II is a widely used 21-item measure with excellent psychometric properties (55), and also validated in Spanish. Cronbach’s α in the pilot sample was 0.90 in adolescents and 0.89 in adults. Alcohol use will be assessed using the Alcohol Use Disorder Identification Test (AUDIT) and drug use will be assessed using the Drug Use Disorder Identification Test (DUDIT). Other symptom rating scales include the Borderline Evaluation of Severity Over Time (BEST) and the Columbia Impairment Scale (CIS). All are self-reports and assessments will be administered at baseline, 3-, 6-, and 12-months f/u.

Implicit Positive and Negative Affect. A dot probe task will assess for attention bias toward negative or positive stimuli (56). The task will comprise 256 very brief trials (approximately 10 minutes total time) consisting of the various combinations of probe type (the letter E or F), probe position (top or bottom), and emotion type (Neutral, Positive, Negative). The stimuli used for this task will be from the same sets of standardized sets of emotional and neutral words used in our pilot project. During the task, gaze will be monitored using a Tobii Pro Nano mobile eye tracker. Each trial begins with a fixation cross that remains on the screen until the participant makes a fixation on the cross. Then, an emotional-neutral word pair is shown on the screen for 500 ms. After this, a probe (E or F) appears in the location of one of the words. The probe remains on the screen until the participant identifies which letter appeared and presses the corresponding button on the keyboard. To facilitate comparison with previous research, we will compute standard attention bias scores reflecting the difference in reaction times to probes following emotional versus neutral words. However, given concerns about reaction time-based indices of attentional bias (57-60), our primary analyses will be indices of initial orienting (location and latency of first fixation) and sustained attention (overall gaze duration) derived from eye-tracking, which have stronger psychometric properties (58, 59). We will also assess attention to PA using the IPANAT (49). This is an indirect assessment of automatic activation of affective representations that utilizes artificial words paired with positive and negative words. Factor analyses of ratings yield two independent factors interpreted as positive and negative. A single score of implicit affect (positive, negative) reflects both state and trait variance. The IPANAT has demonstrated strong convergent and discriminant validity, and adequate internal consistency and test-retest reliability. A series of experiments found a strong trait component (r = .60) but also susceptibility to environmental changes (e.g., affect induction) and is strongly associated with transient explicit PA and NA. Our two proposed tasks to be administered at baseline, 3-, and 6-month f/u, can assess our primary mechanism of PA, as well as our secondary mechanism of NA.

Subjective Positive and Negative Affect. Self-report ratings of PA and NA will be assessed at baseline, 3-, 6-, and 12-month f/u, through the *Modified Differential Emotions Scale* (mDES)(25). The 19-item mDES assesses short-term state positive and negative emotions, along a 5-point Likert scale. Each item consists of three related terms to describe a particular discrete emotion (e.g., glad, happy, joyful). Participants will be asked to rate each set of emotions based on how they were feeling “right now.” The Positive Emotions subscale is a composite of 10 items, while the Negative Emotions subscale is a composite of 9 items. The scale has psychometric support (25) and in the pilot sample, Cronbach’s α was = .86 and .92 for the positive and negative emotions subscales, respectively. To obtain subjective indices of eudemonic (i.e., sustainable) positive affect, we will administer the *Gratitude Questionnaire* (*GQ6*) (61, 62) and the *Satisfaction with Life Scale* *(SWLS)* (63). Both scales use a 7-point Likert response format and will be administered only to the adolescent.

Ancillary Treatment. To assess ancillary treatment, we will administer the Child and Adolescent Services Assessment Screen (CASA) which is a self-report measure at 3-, 6-, and 12-month follow-up, and administer the Treatment History Interview and the Medication Compliance Questionnaire (MCQ), both interviews, at baseline and 3-, 6-, and 12-month follow-up.

Implementation. Implementation Evaluation is based on Proctor et al.’s (64) categorization of implementation outcomes. This study will measure: 1) acceptability, or the extent to which adopting STEP is agreeable, palatable, or satisfactory among key stakeholders, 2) appropriateness, the perceived fit, relevance or compatibility of STEP for a given setting, provider or family; and 3) feasibility, the extent to which STEP can be successfully used or carried out within a given setting. These will be examined using the Acceptability of Intervention Measure (AIM), Intervention Appropriateness Measure (IAM), and the Feasibility of Intervention Measure (FIM), after all post-treatment data have been collected (65). Each are 4-item scales that have demonstrated good psychometric properties including good test-retest reliability and discriminant validity (65).

In addition we will supplement these measures with qualitative interviews with key stakeholders, until thematic saturation is reached (66), to further explore potential barriers to use of STEP in preparation for a future hybrid effectiveness-implementation trial. As part of study participation, patients and the providers are approached about taking part in an exit interview about the STEP program at the end of their participation at the 12-month follow-up. For those who participated in the pre-implementation interviews, they consented to the exit interview at that time. For stakeholders who only participate in the exit interviews, they will sign a consent to participate in the exit interview. The same consenting process will be used with frontline staff who will be approached either at the end of their participation in the trial (e.g. they take a new position), or after the last participant assigned to them to receive the STEP protocol. Persons in leadership positions will be approached in Year 4 after the last randomized participant has completed the STEP intervention. Dr. Elwy will supervise the Research Assistants, who will conduct interviews at the conclusion of the intervention with a variety of stakeholders including 15-20 patients, 15-20 parents, 8-10 front line providers who participated in STEP, and 8-10 people serving in clinical and operational leadership positions. Additionally, after each taped interview, field notes will be written to capture other important aspects of the interview. NVivo will be used for the analyses (67).

Data from the survey measures will provide descriptive information on stakeholders’ perspectives and will be integrated with qualitative data, for an exploratory sequential mixed methods analysis of implementation outcomes resulting from the STEP trial (68). Our qualitative data analysis will involve a directed content analysis approach (69) constructed from an *a priori* framework of codes originating from the Proctor Implementation Outcomes Framework (64). Each set of notes will be analyzed by Dr. Elwy and one RA independently and then compared, as currently used in one of Dr. Spirito’s current studies (NIMH R34 MH113598). This early coding will be presented at a research team meeting with subsequent discussions as to whether specific passages from the interview notes represent the concepts of implementation outcomes, as defined by Proctor et al., or other emergent concepts. Consensus on the reliability and validity of this coding will be achieved through this discussion. The analytic team will then proceed to code the remaining interview notes, with discussion and consensus after every coding completion.

## Safety Assessments

We do an assessment of suicidal ideation and behavior at every assessment time-point as these are our study outcomes. We do not utilize any other safety assessments. If the adolescent endorses suicidal ideation with intent and/or plan within the prior 2 weeks on the C-SSRS, then the RA contacts the clinician who conducts the suicidality procedures described below (See Appendix for full details).

**What is the degree of suicide risk?** Determine the extent of suicidality by asking about ideation, method/plan, the presence of threats (e.g. suicide note) and actual behaviors. If they endorse suicidal ideation, determine the risk that patient will move from low to higher risk. If an attempt has already occurred, determine its nature and assess future risk.

What is your urge to harm yourself right now?

What is your intent to kill yourself right now?

How often do you think about suicide?

How well can you control your thinking about suicide right now?

How well can you control your emotions right now?

How well can you control your actions and behavior right now?

1. **Irrespective of intent, what is preferred method for suicide?** What is the lethality and where will the method occur. i.e., will the person be isolated or is there potential for discovery?
2. **Suicidal intent?** Ask specifically about whether the patient has a desire to die. If yes, consider the degree and type of motivation: actually wants to die, does not particularly want to die but rather wants to escape an intolerable situation or wants to communicate something, such as asking for help, getting attention, making someone feel sorry or change their mind.
3. **Use of alcohol and other drugs**
4. **How effective is the environment for keeping the patient safe?**
5. **Complete safety plan if person will remain in the community.** If assessment indicates an emergency evaluation is necessary, make a plan with the parent and arrangements for an ambulance to transport the youth to the ED.

## Adverse Events and Serious Adverse Events

### Definition of Adverse Events

This protocol uses the definition of adverse event from 21 CFR 312.32 (a): any untoward medical occurrence associated with the use of an intervention in humans, ***whether or not considered intervention-related***. Expected adverse events in this study include NSSI that does not result in hospitalization and breach of confidentiality.

### Definition of Serious Adverse Events

The outcome points for this study include suicidal behavior and hospitalizations/ED visits for suicidal ideation; these have not been considered serious adverse events with suicidal individuals per past IRB guidance. Nonetheless, we will record suicidal behavior and psychiatric ED/inpatient hospitalizations due to suicidal ideation or suicidal behavior as expected SAEs, as well as psychiatric ED visit or inpatient hospitalization not due to SI or a suicide attempt. Given the nature of the population, suicide deaths, although rare in this age group, will also be considered expected SAEs.

The following guidelines will be used to describe severity.

- **Mild** – Events require minimal or no treatment and do not interfere with the participant’s daily activities.
- **Moderate** – Events result in a low level of inconvenience or concern. Moderate events may cause some interference with functioning.
- **Severe** – Events interrupt a participant’s usual daily activity and may require emergency or other treatment. Severe events are usually potentially life-threatening or incapacitating.

#### Relationship to Study INTERVENTION/Experimental Manipulation

All adverse events (AEs) will have their relationship to study procedures, including the intervention, assessed by the project Safety Officer, based on temporal relationship and his/her clinical judgment. The degree of certainty about causality will be graded using the categories below.

- **Definitely Related** – There is clear evidence to suggest a causal relationship, and other possible contributing factors can be ruled out. The clinical event occurs in a plausible time relationship to study procedures administration and cannot be explained by concurrent psychiatric presentation or other factors, such as drugs or chemicals. The response to withdrawal of the study procedures should be clinically plausible. The event must be phenomenologically definitive.
- **Probably Related** – There is evidence to suggest a causal relationship, and the influence of other factors is unlikely. The clinical event occurs within a reasonable time after administration of the study procedures, is unlikely to be attributed to concurrent psychiatric symptoms or other drugs or chemicals, and follows a clinically reasonable response on withdrawal.
- **Potentially Related** – There is some evidence to suggest a causal relationship (e.g., the event occurred within a reasonable time after administration of study procedures). However, other factors may have contributed to the event (e.g., the participant’s clinical condition, other concomitant events). Although an AE may rate only as “possibly related” soon after discovery, it can be flagged as requiring more information and later be upgraded to “probably related” or “definitely related”, as appropriate.
- **Unlikely to be related** – A clinical event whose temporal relationship to study procedures administration makes a causal relationship improbable (e.g., the event did not occur within a reasonable time after administration of the study procedures) and in which other drugs or chemicals or associated psychiatric symptoms provides plausible explanations (e.g., the participant’s clinical condition, other concomitant treatments).
- **Not Related** – The AE is completely independent of study procedures administration, and/or evidence exists that the event is definitely related to another etiology. There must be an alternative, definitive etiology documented by the clinician.

#### Expectedness

The PIs will review every adverse event (AE) and make their own determination regarding whether it is expected or not and related to the study. Our Safety Officer, a clinical psychologist, independent of the study team, with appropriate expertise in suicidal behaviors will be responsible for the ultimate decision of whether an AE is expected or unexpected, and related to the study. An AE will be considered unexpected if the nature, severity, or frequency of the event is not consistent with the risk information previously described for the study procedures.

Given the target recruitment population (adolescents at risk for suicide) and the primary outcomes of suicidal events and ideation, most AEs of this nature will be expected events.

### Time Period and Frequency for Event Assessment and Follow-Up

The occurrence of an adverse event (AE) or serious adverse event (SAE) may come to the attention of study personnel during study visits: baseline, follow-up assessments as well as intervention sessions.

All AEs, will be captured on the appropriate case report form (CRF). Information to be collected includes event description, time of onset, clinician’s assessment of severity and expectedness, relationship to study procedures (assessed only by those with the training and authority to make a diagnosis), and time of resolution/stabilization of the event. All AEs occurring while on study will be documented appropriately regardless of relationship. All AEs will be followed to adequate resolution.

Any medical or psychiatric condition that is present at the time that the participant is screened will be considered as baseline and not reported as an AE.

Changes in the severity of an AE will be documented to allow an assessment of the duration of the event at each level of severity to be performed. Documentation of onset and duration of each episode will be maintained for AEs characterized as intermittent.

Study personnel will record events with start dates occurring any time after informed consent is obtained until 7 (for non-serious AEs) or 30 days (for SAEs) after the last day of study participation. At each study visit, the investigator will inquire about the occurrence of AE/SAEs since the last visit. Events will be followed for outcome information until resolution or stabilization.

### Adverse Event Reporting

Once staff members report an AE to MPIs, Drs. Spirito or Yen will code severity, causal relationship, and whether the event was expected. If necessary, they will seek further information from the participant or parent or other source before coding. AEs will be coded on a weekly basis.

Any potential SAE will be internally reviewed (typically within 24 hours) by one of the MPIs (or a qualified designee in their absence) and independently will be reviewed by the Safety Officer at the same time. Coding of AEs and SAEs occurs on a study AE case report form. In the case of disagreement, the PIs and SO will discuss, but the SO’s determination will be adopted.

### Serious Adverse Event Reporting

As noted above, in addition to the review by the MPIs, the independent Safety Officer will be responsible for conducting an evaluation of serious adverse events. SAE reports will be provided to the IRB and NIMH DSMB according to the timelines listed in Table 1. SAEs that are unanticipated, serious, and possibly related to the study intervention will be reported on an expedited timeline (5 business days) to the DSMB and IRB, in accordance with requirements. Deaths (by suicide or other causes) will be reported within 5 days to the IRB and NIMH DSMB.

Expected or unrelated SAEs will be reported in a less urgent manner to the IRB and the NIMH DSMB on the reporting timeline included in Table 1; AEs will be reported to the NIMH DSMB on an annual basis.

## Unanticipated Problems

### Definition of Unanticipated Problems

This protocol uses the definition of Unanticipated Problems as defined by the Office for Human Research Protections (OHRP). OHRP considers unanticipated problems involving risks to participants or others to include, in general, any incident, experience, or outcome that meets **all** of the following criteria:

- Unexpected in terms of nature, severity, or frequency given (a) the research procedures that are described in the protocol-related documents, such as the Institutional Review Board (IRB)-approved research protocol and informed consent document; and (b) the characteristics of the participant population being studied;
- Related or possibly related to participation in the research (“possibly related” means there is a reasonable possibility that the incident, experience, or outcome may have been caused by the procedures involved in the research); and
- Suggests that the research places participants or others at a greater risk of harm (including physical, psychological, economic, or social harm) than was previously known or recognized.

### Unanticipated Problems Reporting

The investigators and Safety Officer will review all unanticipated problems (UPs) and report them to the reviewing IRB and to the DSMB. The UP report will include the following information:

- Protocol identifying information: protocol title and number, PI’s name, and the IRB project number
- A detailed description of the event, incident, experience, or outcome
- An explanation of the basis for determining that the event, incident, experience, or outcome represents an UP
- A description of any changes to the protocol or other corrective actions that have been taken or are proposed in response to the UP

To satisfy the requirement for prompt reporting, UPs will be reported using the following timeline:

- UPs that are serious adverse events (SAEs) will be reported to the IRB and DSMB, and to the study sponsor/funding agency within 5 working day of the investigators becoming aware of the event.
- Any other UP will be reported to the IRB and DSMB within 5 business days of the investigator becoming aware of the problem and reviewing with the Safety Officer.
- All UPs should be reported to appropriate institutional officials (as required by an institution’s written reporting procedures), NIMH, and the Office for Human Research Protections (OHRP) within 5 days of the IRB’s receipt of the report of the problem from the investigator.

*See* ***CD Section 8.4.1*** *for additional example text applicable for devices.*

# STATISTICAL CONSIDERATIONS

**Data Analyses.** In this section, we describe aim by aim the proposed method of data analysis and sample size considerations. **Scientific rigor** is enhanced and maintained by: 1) randomization procedures, 2) having assessment staff blind to condition, 3) multiple modalities of assessment, 4) comprehensive retention procedures, 5) preliminary analyses, 6) missing data procedures, and 7) data analytic strategies that are consistent with best practices for the analysis of randomized clinical trials as embodied in the Consolidated Standards of Reporting Trials (CONSORT) statement (75)**.** Additionally, relevant biological variables, specifically sex, are considered in the experimental design. We do not have a priori hypotheses regarding sex differences in the effect of STEP on our study outcomes. Accordingly, and in agreement with NIH guidance on this topic our plan is to separately tabulate results by sex but test our main hypotheses in a sample with both sexes combined (<http://orwh.od.nih.gov/sexinscience/overview/pdf/NOT-OD-15-102_Guidance.pdf>). Finally, by collecting implementation outcome data, we will be able to identify and develop implementation strategies to encourage greater uptake and adoption of STEP in a future R01 implementation trial. As all our hypotheses are pre-specified, no adjustment for multiplicity will be used.


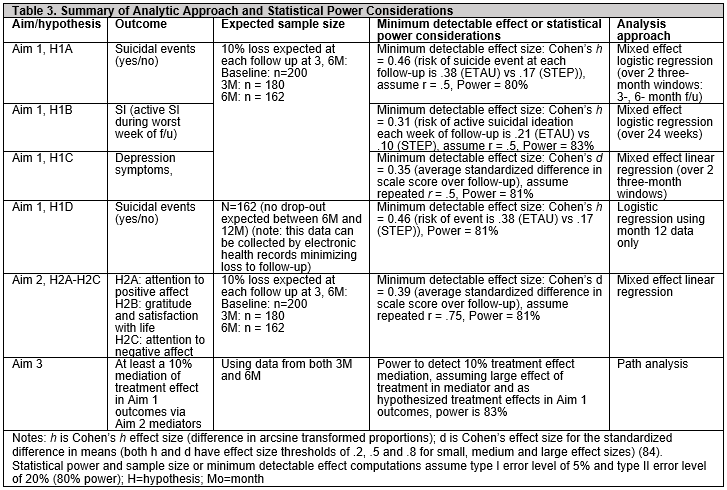
Our randomization procedures include stratification on important predictors of the main outcome variable and are automated and protected from inadvertently revealing treatment assignment order to staff. This will help ensure the blinding of assessment staff to treatment condition. Our plan for how missing data will be

handled with multiple imputations, with assumptions checked with sensitivity analyses (76), is pre-specified (as are all aspects of our analysis plan). We will adhere to intent-to-treat principles, and all aspects of data analysis will be pre-specified as reflected in this proposal, and with modification following proposal review and discussions with program and our Data Safety and Monitoring Board. Preliminary analyses will include descriptive statistics to examine the distributional and psychometric properties of the variables (e.g., normality, internal consistency). Variables will be transformed to better approximate normality if this is necessary given the observed distribution and the assumptions of the analysis model. We will also examine post-inclusion attrition by comparing study completers to dropouts on sociodemographic, baseline data, and psychotropic medication usage to determine if they differ systematically. We will examine the success of the stratified randomization by examining between-group differences at baseline. Preliminary analyses may also include analyses of adverse events, the progress of recruitment and retention, and data processing and quality markers as the study progresses. Daily mood monitoring data is not included in outcome analyses because it is conceptualized as part of this adjunctive intervention and only collected in one condition.

Missing data. To accommodate attrition and other sources of missingness, we will utilize the method of multiple imputation by chained equations. At least 50 replicates of each missing value will be generated for inclusion in analyses. This method can accommodate clustering of repeated measures at the level of the. We have previously used these methods in successful analyses of trials of exercise interventions.

Statistical computing. Modeling will be performed using Stata (Stata Corp, College Station, TX, version 16.2 or higher) and R software (R Foundation for Statistical Computing, Vienna, Austria, version 4.0 or higher) and Mplus (version 8.4 or higher, Muthen & Muthen, Los Angeles, CA). Analyses and reporting will be executed and archived to ensure full replicability and reproducibility by analysts at Brown University, under the direction of the project methodologist, Dr. Jones.

**Aim 1A-C:** *Examine the effectiveness of STEP in reducing suicidal events, active SI*, *and depression at 3- and 6-month follow-up (f/u).* We hypothesize that those randomized to STEP, compared to ETAU, will have (**H1A**) lower prevalence of suicidal events (operationalized as either a suicide attempt or emergency intervention to intercede when there is a high likelihood of an attempt) **(H1B**) lower prevalence of time with active SI (operationalized as SI with intent or plan), and (**H1C**) lower level of depressive symptoms, over the 6-month follow-up period. Aim 1 analyses will use a repeated measures design and a multilevel or mixed effect modeling framework. For H1A and H1B, the outcome is binary and a mixed effect logistic regression model will be used. For H1C, the outcome is continuous and a mixed effect general linear model will be used. For H1B, the outcomes are repeatedly observed over follow-up weeks within the 6M timeframe (24 weeks), and can yield a categorical (active SI, worst week) outcome, which would be analyzed like H1A or a continuous (proportion of weeks with active SI) outcome, which would be analyzed differently like H1C. For H1A and H1C the outcome is observed twice at the scheduled follow-ups (3M, 6M). For all hypotheses, we will treat the individual as the random effect. Treatment effects will be estimated as the weighted average of the differences between ETAU and STEP groups over all follow-up times, adjusting for follow-up time.

In secondary analyses to identify potential mechanisms (i.e. mediators) of treatment effectiveness that occur after randomization, we will repeat these analyses using medication use and psychosocial treatment as covariates.

In secondary analyses, payor data will be examined as a potential moderator or predictor of these outcomes.

In secondary analyses, the patterns of change in depressed mood (BDI scores) will be evaluated using random effects models for longitudinal data.

***Sample size and statistical power considerations:*** We determined minimal detectable effect sizes using Monte Carlo methods. This involved generating 1,001 simulated data sets representing our hypothesized effects, and our assumptions of a 10% loss by 3M and another 10% loss by 6M follow-up. We assumed repeated measures of depressive symptoms and the propensity to active suicidal ideation and suicidal events were all correlated at *r* = .5. Across the simulated data sets, we compute power as the proportion of replicates returning a significant effect in the hypothesized direction. Results of these models are summarized in Table 3, above. We summarize effects in terms of Cohen’s effect size statistics for clear expression of the minimum detectable effects. For H1A, H1B, and H1C, we will have sufficient power to detect small-to-medium effect sizes (.46, .31, .35, respectively).

**Aim 1D:** *Examine the effectiveness of STEP in reducing suicidal events at the 12-month f/u.* We hypothesize that those randomized to STEP, compared to ETAU, will have lower rates of suicidal events at the 12-month, long term f/u. This hypothesis will be tested to evaluate the durability of STEP effects over long-term follow-up. We expect to see the full effect of STEP at the 6M follow-up. This hypothesis will be evaluated with logistic regression, and not as part of the repeated measures analysis specified in H1A. If the effect continues through 12M, we will have 80% power to detect a difference in event rates of 38% ETAU and 17% STEP at the 12M follow-up.

In secondary analyses, the within-person frequency of suicidal events and suicidal ideation will be analyzed. These analyses will use (zero inflated) negative binomial regression models to account for the anticipated extreme skew of these outcomes.

***Sample size and statistical power considerations***. The power to test the difference between two proportions, .17 and .38, is significantly different from zero can be found from POWER = F(sqrt(((n*(p1-p2)^2)/(p0*(1-p0)))/2) – F’(1-(a/2))), where F is the cumulative normal density function and F’ is the normal quantile function, a is the desired type-I error level, n is the per-group sample size and p1 and p2 are the target proportions. H1A and H1D have similar minimum detectable effect sizes because we use a conservative estimate of the within-person correlation of propensity to suicide for aims H1A-H1C. We conservatively use 162 as our sample size for these calculations but we expect to have a larger sample with suicidal event data because these data can be drawn from the electronic health record in addition to participant self-report.

**Aim 2:** *Examine engagement of the hypothesized mechanisms at the 3- and 6-month f/u.* We hypothesize that those randomized to STEP, compared to ETAU, will have*:* **H2A:** higher attention to positive affect, assessed by implicit tasks; **H2B**: Increased gratitude and satisfaction with life, assessed by self-report; **H2C**: lower attention to negative affect, assessed by implicit tasks and self-report. Aim 2 analyses will use a repeated measures design and a multilevel or mixed effect modeling framework. For H2A, H2B, and H2C, the outcomes are continuous and a mixed effect general linear model will be used. Outcomes are repeatedly observed over follow-up and assessed at the scheduled follow-up (3M, 6M). For all hypotheses, we will treat the individual as the random effect. Treatment effects will be estimated as the weighted average of the differences between ETAU and STEP groups overall follow-up times, adjusting for follow-up time. ***Sample size and statistical power considerations***: As with H1, we determined minimal detectable effect sizes using Monte Carlo methods. This involved generating 1,001 simulated data sets representing our hypothesized effects, and our assumptions of a 10% loss by 3M and another 10% loss by 6M follow-up. We assumed repeated measures of the measures assessing the mechanisms (attention to positive affect, gratitude and satisfaction with life, and attention to negative affect) to have a stronger correlation within a respondent over time than the Aim 1 outcomes, and assumed *r* = .75, which results in a smaller effective sample size. Across the simulated data sets, we compute power as the proportion of replicates returning a significant effect in the hypothesized direction. Results of these models are summarized in Table 3, above. We summarize effects in terms of Cohen’s effect size statistics for clear expression of the minimum detectable effects. For Aim 2 outcomes, we will have sufficient power to detect small-to-medium effect sizes (*d* = .39).

**Aim 3**: *Examine whether hypothesized mechanisms mediate reduction of suicidal events and ideation*. **H3:** Treatment-related changes in attention to positive affect, gratitude, satisfaction with life, and attention to negative affect at 3M follow-up will be related to improvements in SE and SI at the 6M f/u. Analysis: Mediation will occur when the outcomes (SE, SI) and the putative mediators (PA, gratitude and satisfaction with life, NA) are correlated, and that the distribution of the mediators varies according to treatment (Aim 2). We will approach the analysis using the Baron & Kenney (77) approach to mediation, with modifications for non-normal dependent variables, which is similar to the principal stratification approach to causal mediation analysis (78). We will approach this analysis using a cross-lagged panel model and a path analysis framework. Specifically, we will regress 6M outcomes and mediators on 3M outcomes and 3M mediators, and evaluate the effect of 6M outcomes regressed on 3M mediators accounting for any residual correlation of 6M outcome and 6M mediators and the 3M correlation of outcomes and mediators. All outcomes and mediators at all time points are regressed on treatment condition. We will quantify the total effect of treatment assignment in 6M outcomes, and the indirect effect of treatment in 6M outcomes via M3 mediators. Following Mandolando and Greenland (79), we will infer meaningful level of mediation if at least 10% of the total effect of treatment in 6M outcomes is mediated via M3 mediators. We will characterize the precision and statistical significance of mediation effects using bootstrap methods for interval estimation (80). ***Sample size and statistical power considerations.*** We used Monte Carlo methods to identify conditions in which a 10% difference (or greater difference) in the main treatment effect is to be detected when comparing to a model adjusting for the putative mediator. Finding a mediation effect requires that the treatment group have a different distribution on the putative mediator (attention to positive affect, gratitude and satisfaction with life, attention to negative affect) and the mediator be related to the outcomes (SE, active SI). In plausible scenarios (a unit standard deviation difference on the mediator across treatment group), and a (polyserial) correlation of 0.3 or greater between the mediator and the outcome, we will have adequate (at least 88%) power to detect a 10% mediated effect of treatment group and the outcome (n = 162, 81 per group). Similarly, if the treatment produces a net half standard deviation difference in the mediator (reflecting a clinically meaningful change), and the mediator has a (polyserial) correlation of at least .25 with the outcome, we will have 83% power to detect the target mediated effect.

# SUPPORTING DOCUMENTATION AND OPERATIONAL CONSIDERATIONS

## Regulatory, Ethical, and Study Oversight Considerations

### Informed Consent Process

#### Consent/assent and Other Informational Documents Provided to participants

Consent and assent forms describing in detail the study intervention, study procedures, and risks will be given to the participant and (if under 18) their guardian. If a participant turns 18 during the course of the study, they will be re-consented with the Consent Form. Stakeholder consents contain this same information and written documentation of informed consent will be completed prior to starting the study intervention for both participants and stakeholders. The following consent materials are submitted with this protocol:

- Consent form for participant (age 18)
- Parent permission form for participant (under age 18)
- Assent form
- Consent form for parent
- Consent form for Stakeholders, who include clinicians, administrators

#### Consent Procedures and Documentation

Our team has developed effective procedures for recruiting; study research assistants will screen for eligibility through review of admission notes to identify participants hospitalized due to suicide risk. They will then obtain approval from clinical staff at Butler Hospital, Bradley Hospital, and the BCH to approach the participant to ascertain interest and confirm that the patient does not have psychotic thoughts and has the cognitive capacity to understand the study. If interested, research staff will obtain informed parent consent as well as adolescent assent and schedule a baseline assessment to be conducted on the unit, which will include confirmation of suicide risk with the C-SSRS. Additionally, if adolescents are interested in participating in the study, but parents are not, adolescents will be allowed to participate with their parent’s consent.

Parents who are interested in the study will be contacted to set up a consenting call to allow ample time to explain study details and answer any questions. Prior to the consenting call, the RA will create a record ID in REDCap and electronically send a link to the appropriate consent documents to the parent/guardian. The RA will also confirm receipt of this link. At the time of the call, the RA will ask the parent to open the REDCap link and will guide them through the consent form and answer questions as they arise. The REDCap consent documents have been set to include “required responses” in all sections where participants must opt in or out of audio recording, different avenues of contact, and the NIMH NDA, as well as provide their initials to confirm these choices before they can move onto the next section. This is done to minimize the possibility of completing the forms partially or incorrectly. The RA will guide the parent on where to sign and date the document before submitting. Once the consent document is completed, the RA will review the consent form for completion or errors. If any mistakes are found, the RA will prompt the parent to revise those errors by returning to the consent document using the same link. Once the consent process is finalized, a second research team member will review the consent form to verify that it was completed correctly before the study commences with the participant. The secondary check will be noted in our internal tracking log.

For adolescent assent, the RA will meet with the teen in-person on the unit to complete the electronic assent form. The procedures for information verification and secondary quality assurance checks are the same as above.

If a parent is not fluent in English but they are fluent in Spanish, we will have Spanish consent forms and obtain consent with a native Spanish speaker, typically an RA, or utilize translator services at each respective hospital, if necessary, in order to obtain informed consent. We will use Spanish language versions of self-report forms where available, and translate those that are not available in Spanish.

### Study Discontinuation and Closure

This study may be temporarily suspended or prematurely terminated if there is sufficient reasonable cause. Written notification, documenting the reason for study suspension or termination, will be provided by the suspending or terminating party to study participants, investigator, funding agency, and regulatory authorities. If the study is prematurely terminated or suspended, the MPIs will promptly inform study participants, the Institutional Review Board (IRB), DSMB, and sponsor/funding agency and will provide the reason(s) for the termination or suspension. Study participants will be contacted, as applicable, and be informed of changes to study visit schedule.

Circumstances that may warrant termination or suspension include, but are not limited to:

- Determination of unexpected, significant, or unacceptable risk to participants
- Insufficient compliance to protocol requirements
- Data that are not sufficiently complete and/or evaluable
- Determination of futility

The study may resume once concerns about safety, protocol compliance, and data quality are addressed, and satisfy the funding agency, sponsor, IRB, and other relevant regulatory or oversight bodies (Safety Officer, DSMB).

### Confidentiality and Privacy

Participant confidentiality and privacy is strictly held in trust by the participating investigators, their staff, the safety and oversight monitor(s), and the sponsor(s) and funding agency. This confidentiality is extended to the data being collected as part of this study. Data that could be used to identify a specific study participant will be held in strict confidence within the research team. No personally-identifiable information from the study will be released to any unauthorized third party without prior written approval of the sponsor/funding agency.

Limits of confidentiality are restricted to concerns about safety to self and others. Parents will not have access to their child’s messages, and none of the messages are intended to assess safety risk. Reports of abuse or neglect will be investigated further. Should a participant disclose abuse, neglect, intent to harm someone else, etc., a hotline call and report will be made to the appropriate child and family agency within 24 hours.

All research activities will be conducted in as private a setting as possible.

The Safety Officer Sarah Arias, PhD, other authorized representatives of the sponsor or funding agency, representatives of the Institutional Review Board (IRB), regulatory agencies, may inspect all documents and records required to be maintained by the investigator, including but not limited to, medical records for the participants in this study. The clinical study site will permit access to such records.

The study participant’s contact information will be securely stored at each clinical site for internal use during the study. At the end of the study, all records will continue to be kept in a secure location for as long a period as dictated by the reviewing IRB, Institutional policies, or sponsor/funding agency requirements.

Study participant research data, which is for purposes of statistical analysis and scientific reporting, will be transmitted to and stored at the Data Coordinating Site, based in the Lifespan Healthcare System and Brown University. This will not include the participant’s contact or identifying information. Rather, individual participants and their research data will be identified by a unique study identification number. The study data entry and study management systems used by clinical sites and by data coordinating site research staff will be secured and password protected. At the end of the study, all study databases will be de-identified and archived at the data coordinating center.

Measures Taken to Ensure Confidentiality of Data Shared per the NIH Data Sharing Policies

It is NIH policy that the results and accomplishments of the activities that it funds should be made available to the public (see <https://grants.nih.gov/policy/sharing.htm>). The PI will ensure all mechanisms used to share data will include proper plans and safeguards for the protection of privacy, confidentiality, and security for data dissemination and reuse (e.g., all data will be thoroughly de-identified and will not be traceable to a specific study participant). Plans for archiving and long-term preservation of the data will be implemented, as appropriate.

**Certificate of Confidentiality**

To further protect the privacy of study participants, the Secretary, Health and Human Services (HHS), has issued a Certificate of Confidentiality (CoC) to all researchers engaged in biomedical, behavioral, clinical or other human subjects research funded wholly or in part by the federal government.  Recipients of NIH funding for human subjects research are required to protect identifiable research information from forced disclosure per the terms of the NIH Policy (see <https://humansubjects.nih.gov/coc/index>). As set forth in [45 CFR Part 75.303(a)](https://www.ecfr.gov/cgi-bin/text-idx?SID=f3e9328bbbd5aabe8e639ca48dcbcc7f&mc=true&node=se45.1.75_1303&rgn=div8) and [NIHGPS Chapter 8.3](https://grants.nih.gov/grants/policy/nihgps/HTML5/section_8/8.3_management_systems_and_procedures.htm), recipients conducting NIH-supported research covered by this Policy are required to establish and maintain effective internal controls (e.g., policies and procedures) that provide reasonable assurance that the award is managed in compliance with Federal statutes, regulations, and the terms and conditions of award. It is the NIH policy that investigators and others who have access to research records will not disclose identifying information except when the participant consents or in certain instances when federal, state, or local law or regulation requires disclosure. NIH expects investigators to inform research participants of the protections and the limits to protections provided by a Certificate issued by this Policy.

### Future Use of Stored Specimens and Data

Data collected for this study will be analyzed and stored at the Brown University. The de-identified, archived data will be transmitted to the NIMH National Data Archive (NDA), on a schedule dictated by the NDA, for use by other researchers outside of the study. Permission to transmit data to the NIMH NDA will be included in the informed consent.

When the study is completed, access to study data and/or samples will be provided through the NIMH NDA.

### Key Roles and Study Governance

| **Principal Investigator** | **Principal Investigator** | **Independent Safety Officer** |
| --- | --- | --- |
| *Anthony Spirito, Ph.D.*  *Professor* | *Shirley Yen, Ph.D.*  *Associate Professor* | *Sarah Arias, Ph.D.*  *Assistant Professor (Research)* |
| *Brown University* | *Beth Israel Deaconess Medical Center* | *Butler Hospital* |
| *700 Butler Drive*  *Providence, RI 02906* | *75 Fenwood Road*  *Boston, MA 02115* | *345 Blackstone Blvd,*  *Providence RI, 02906* |
| 401-444-1929 | 617-626-9370 | 401-455-6261 |
| *Anthony_Spirito@Brown.edu* | *Syen1@bidmc.harvard.edu* | *Sarah_Arias@Brown.edu* |

### Safety Oversight

Safety oversight will be under the direction of the NIMH Data and Safety Monitoring Board (DSMB) composed of individuals with the appropriate expertise, including expertise in suicide risk assessment and intervention development. In addition, the study will have a Safety Officer independent from the study conduct and free of conflict of interest. The Safety Officer will meet at least 3X per year to assess aggregate safety and efficacy data from each arm of the study; additional responsibilities are delineated in Section 8.3 regarding safety monitoring. The DSMB will operate under the rules of an approved charter that will be written and reviewed at the organizational meeting of the DSMB. Before the study begins, each data element that the DSMB needs to assess will be clearly defined. The DSMB will provide its input to NIMH.

### Clinical Monitoring

Clinical site monitoring will be conducted to ensure that the rights and well-being of trial participants are protected, that the reported trial data are accurate, complete, and verifiable, and that the conduct of the trial is in compliance with the currently approved protocol/amendment(s), with International Council on Harmonization Good Clinical Practice (ICH GCP), and with applicable regulatory requirement(s). The administrator of the Butler IRB, the IRB of record, will serve as the study monitor.

### Quality Assurance and Quality Control

Each clinical site will perform internal quality management of study conduct, data collection, documentation and completion. All sites will follow a common quality management plan to be developed in advance of study start up.

Quality control (QC) procedures will be reviewed annually and include:

**Informed consent ---** Study staff will review both the documentation of the consenting process as well as a percentage of the completed consent documents. This review will evaluate compliance with GCP, accuracy, and completeness. Feedback will be provided to the study team to ensure proper consenting procedures are followed. Study staff obtaining consent will be required to get a secondary quality assurance check from a different member of the research team prior to working with the participant, to verify completion of every section of the consent document and to review any potential errors. Once the consent is completely and accurately completed, it will be noted in the study tracking system.

**Source documents and the electronic data ---** Data will be initially captured on source documents (see **Section 10.1.9, Data Handling and Record Keeping**) and will ultimately be entered into the study database. To ensure accuracy site staff will compare a representative sample of source data against the database, targeting key data points in that review.

**Intervention Fidelity** — Consistent delivery of the study interventions will be monitored throughout the intervention phase of the study. Procedures for ensuring fidelity of intervention delivery are described in **Section 6.2.1, Interventionist Training and Tracking**.

**Protocol Deviations** – The study team will review protocol deviations on an ongoing basis and will implement corrective actions when the quantity or nature of deviations are deemed to be at a level of concern.

**Text Messaging**. The functioning of the text messaging program will be checked on a monthly basis, with respect to both functionality and data collection.

Should independent monitoring become necessary, the MPIs will provide direct access to all trial related sites, source data/documents, and reports for the purpose of monitoring and auditing by the sponsor/funding agency, and inspection by local and regulatory authorities.

### Data Handling and Record Keeping

#### Data Collection and Management Responsibilities

Data collection will be the responsibility of the clinical trial staff at the site under the supervision of the site investigator. The investigator will be responsible for ensuring the accuracy, completeness, legibility, and timeliness of the data reported.

All source documents will be completed in a comprehensive fashion to ensure accurate interpretation of data.

Hardcopies of the study visit worksheets will be provided for use as source document worksheets for recording data for each participant consented/enrolled in the study. Data recorded in the electronic case report form (eCRF) derived from source documents will be consistent with the data recorded on the source documents.

Clinical data (including adverse events (AEs), concomitant medications, and expected adverse reactions data) and clinical laboratory data will be entered into REDCap, a 21 CFR Part 11-compliant data capture system provided by Lifespan/Brown University. The data system includes password protection and internal quality checks, such as automatic range checks, to identify data that appear inconsistent, incomplete, or inaccurate. Clinical data will be entered directly from the source documents.

#### Study Records Retention

No records will be destroyed without the written consent of the sponsor/funding agency, if applicable. It is the responsibility of the sponsor/funding agency to inform the investigator when these documents no longer need to be retained with the operating assumption being they will be destroyed after 7 years.

### Protocol Deviations

This protocol defines a protocol deviation as any noncompliance with the clinical trial protocol, International Council on Harmonization Good Clinical Practice (ICH GCP), or Manual of Procedures (MOP) requirements. The noncompliance may be either on the part of the participant, the investigator, or the study site staff. As a result of deviations, corrective actions will be developed by the site and implemented promptly; the MPIs will review all protocol deviations and institute corrective actions as appropriate when such deviations and violations occur.

These practices are consistent with ICH GCP:

- Section 4.5 Compliance with Protocol, subsections 4.5.1, 4.5.2, and 4.5.3
- Section 5.1 Quality Assurance and Quality Control, subsection 5.1.1
- Section 5.20 Noncompliance, subsections 5.20.1, and 5.20.2.

It will be the responsibility of the site investigator to use continuous vigilance to identify and report deviations annually in the progress report. All deviations will be addressed in study source documents, reported to NIMH Program Official, the DSMB and the IRB in the annual report (see Table 1). Protocol deviations will be sent to the reviewing Institutional Review Board (IRB) per their policies in the annual report.

Protocol Violations, defined as deviations from the protocol that could result in participant safety concerns, will be

reported to the IRB within 10 days and to the NIMH DSMB at the subsequent meeting. Protocol violations that

threaten the integrity of the study data will be reported to the NIMH DSMB at the subsequent meeting.

The MPIs will review all protocol deviations and institute corrective actions as appropriate when such deviations

and violations occur.

### Publication and Data Sharing Policy

This study will be conducted in accordance with the following publication and data sharing policies and regulations:

National Institutes of Health (NIH) Public Access Policy, which ensures that the public has access to the published results of NIH funded research. It requires scientists to submit final peer-reviewed journal manuscripts that arise from NIH funds to the digital archive PubMed Central upon acceptance for publication.

This study will comply with the NIH Data Sharing Policy and Policy on the Dissemination of NIH-Funded Clinical Trial Information and the Clinical Trials Registration and Results Information Submission rule. As such, this trial will be registered at ClinicalTrials.gov, and results information from this trial will be submitted to ClinicalTrials.gov. In addition, every attempt will be made to publish results in peer-reviewed journals. Data from this study may be requested from other researchers x years after the completion of the primary endpoint by contacting MPI Spirito or MPI Yen. Considerations for ensuring confidentiality of these shared data are described in Section 10.1.3.

### Conflict of Interest Policy

The independence of this study from any actual or perceived influence, is critical. Therefore, any actual conflict of interest of persons who have a role in the design, conduct, analysis, publication, or any aspect of this trial will be disclosed and managed. Furthermore, persons who have a perceived conflict of interest will be required to have such conflicts managed in a way that is appropriate to their participation in the design and conduct of this trial. The study leadership in conjunction with the NIMH has established policies and procedures for all study group members to disclose all conflicts of interest and will establish a mechanism for the management of all reported dualities of interest.

## Abbreviations and Special Terms

All abbreviations and terms have been defined in the body of the text.

## Protocol Amendment History

*The table below is intended to capture changes of IRB-approved versions of the protocol, including a description of the change and rationale. A* ***Summary of Changes*** *table for the current amendment is located in the* ***Protocol Title Page****.*

| **Version** | **Date** | **Description of Change** | **Brief Rationale** |
| --- | --- | --- | --- |
| 1 |  | Original Protocol | NA |
| 2 | 10/07/2022 | To allow remote delivery of messages via email if that is the preferred method; TAU messages to integrate some Healthy Habits messages in addition to reminders of safety plan. | The changes to distribution of the study material is to allow participants who may not have access to their phones after discharge to still participate in the study. For our treatment as usual condition, a mix of healthy habits messages with reminders of safety planning are sent, in order to provide an equivalent amount of contact across conditions. |
| 3 | 01/27/2023 | To increase parent engagement in the study, a handout highlighting study goals, contact information, and ways to help support their child throughout the duration of the study will be provided to parents/guardians after consenting and randomization to the STEP TX condition at the time of discharge. | Due to the nature of the 4 STEP sessions being individual with interventionist and adolescent participant, an effort to increase parent engagement in the study was needed. Increased parent engagement will allow for greater participant retention and more support for the child involved in the study. |
| 3 | 01/27/2023 | Inclusion of language for compressed STEP TX sessions | To accommodate participants with a shorter length of stay in the hospital, some of the 4 individual STEP sessions might be shortened at times to ensure delivery of the intervention prior to discharge. |
| 3 | 03/16/2023 | Inclusion of specific electronic consent procedures detailing the use of REDCap and quality assurance checks. | To minimize errors in consent document completion and increase participant understanding and documentation quality, additional detail has been included to provide further information on specific consent procedures per the IRB’s request. |
| 3 | 03/17/2023 | Addition of language regarding 4 videos containing content from the STEP treatment manual to support the skills covered in the in-person sessions. | As part of the texting intervention, participants receive links to videos to help them practice. We have created 4 brief videos that are based on content from our treatment manual. These videos will be used to support the sessions, and/or will be sent out upon discharge and as part of our remote delivery via text messaging. |
|  |  |  |  |
|  |  |  |  |
|  |  |  |  |
|  |  |  |  |
|  |  |  |  |
|  |  |  |  |
|  |  |  |  |
|  |  |  |  |
|  |  |  |  |
|  |  |  |  |
|  |  |  |  |
|  |  |  |  |
|  |  |  |  |
|  |  |  |  |
|  |  |  |  |
|  |  |  |  |

# REFERENCES

**REFERENCES**

1. Hedegaard H, Curtin SC, Warner M. Suicide mortality in the United States, 1999-2017: US Department of Health and Human Services, Centers for Disease Control and …; 2018.

2. Curtin SC, Warner M, Hedegaard H. Increase in suicide in the United States, 1999–2014. NCHS data brief. 2016;241:1-8.

3. Kann L, McManus T, Harris W, Shanklin S, Flint K, Hawkins J, Zaza S. Youth risk behavior surveillance-United States, 2015. MMWR Surveillance Summaries, 65 Suppl 6, 1-174. 2016.

4. Plemmons G, Hall M, Doupnik S, Gay J, Brown C, Browning W, Casey R, Freundlich K, Johnson DP, Lind C. Hospitalization for suicide ideation or attempt: 2008–2015. Pediatrics. 2018;141(6):e20172426.

5. Appleby L, Morriss R, Gask L, Roland M, Lewis B, Perry A, Battersby L, Colbert N, Green G, Amos T, Davies L, Faragher B. An educational intervention for front-line health professionals in the assessment and management of suicide patients (The STORM Project). Psychological Medicine. 2000;30(4):805-12.

6. Goldston DB, Daniel SS, Reboussin DM, Reboussin BA, Frazier PH, Kelly AE. Suicide attempts among formerly hospitalized adolescents: A prospective naturalistic study of risk during the first 5 years after discharge. Journal of the American Academy of Child and Adolescent Psychiatry. 1999;38(6):660-71.

7. Prinstein MJ, Nock MK, Simon V, Aikins JW, Cheah CS, Spirito A. Longitudinal trajectories and predictors of adolescent suicidal ideation and attempts following inpatient hospitalization. J Consult Clin Psychol. 2008;76(1):92-103. Epub 2008/01/31. doi: 2008-00950-012 [pii] 10.1037/0022-006X.76.1.92 [doi]. PubMed PMID: 18229987.

8. Hunt IM, Kapur N, Webb R, Robinson J, Burns J, Shaw J, Appleby L. Suicide in recently discharged psychiatric patients: a case-control study. Psychological medicine. 2009;39(3):443-9.

9. Yen, Weinstock L, Andover M, Sheets E, Selby E, Spirito A. Prospective predictors of adolescent suicidality: 6-month post-hospitalization follow-up. Psychological medicine. 2013;43(05):983-93.

10. Esposito-Smythers C, Wolff JC, Liu RT, Hunt JI, Adams L, Kim K, Frazier EA, Yen S, Dickstein DP, Spirito A. Family-focused cognitive behavioral treatment for depressed adolescents in suicidal crisis with co-occurring risk factors: a randomized trial. J Child Psychol Psychiatry. 2019. Epub 2019/07/23. doi: 10.1111/jcpp.13095. PubMed PMID: 31328281.

11. Knesper DJ. Continuity of care for suicide prevention and research: suicide attempts and suicide deaths subsequent to discharge from an emergency department or an inpatient psychiatry unit: Suicide Prevention Resource Center; 2011.

12. James S, Charlemagne SJ, Gilman AB, Alemi Q, Smith RL, Tharayil PR, Freeman K. Post-discharge services and psychiatric rehospitalization among children and youth. Administration and Policy in Mental Health and Mental Health Services Research. 2010;37(5):433-45.

13. Fontanella CA. The influence of clinical, treatment, and healthcare system characteristics on psychiatric readmission of adolescents. American Journal of Orthopsychiatry. 2008;78(2):187-98.

14. O'Connor E, Gaynes BN, Burda BU, Soh C, Whitlock EP. Screening for and treatment of suicide risk relevant to primary care: a systematic review for the US Preventive Services Task Force. Annals of internal medicine. 2013;158(10):741-54.

15. Tarrier N, Taylor K, Gooding P. Cognitive-behavioral interventions to reduce suicide behavior: a systematic review and meta-analysis. Behav Modif. 2008;32(1):77-108. Epub 2007/12/22. doi: 32/1/77 [pii] 10.1177/0145445507304728 [doi]. PubMed PMID: 18096973.

16. Esposito-Smythers C, Spirito A, Kahler CW, Hunt J, Monti P. Treatment of co-occurring substance abuse and suicidality among adolescents: a randomized trial. J Consult Clin Psychol. 2011;79(6):728-39. Epub 2011/10/19. doi: 2011-23878-001 [pii] 10.1037/a0026074 [doi]. PubMed PMID: 22004303; PMCID: 3226923.

17. McCauley E, Berk MS, Asarnow JR, Adrian M, Cohen J, Korslund K, Avina C, Hughes J, Harned M, Gallop R. Efficacy of dialectical behavior therapy for adolescents at high risk for suicide: a randomized clinical trial. JAMA psychiatry. 2018;75(8):777-85.

18. Li D, Zhang W, Li X, Li N, Ye B. Gratitude and suicidal ideation and suicide attempts among Chinese adolescents: Direct, mediated, and moderated effects. Journal of adolescence. 2012;35(1):55-66.

19. Hirsch JK, Duberstein PR, Chapman B, Lyness JM. Positive affect and suicide ideation in older adult primary care patients. Psychology and Aging. 2007;22(2):380-5. doi: 10.1037/0882-7974.22.2.380. PubMed PMID: pag-22-2-380. PsycINFO AN: 2007-07952-017. PMID: 17563193. First Author & Affiliation: Hirsch, Jameson K.

20. Fredrickson BL. The role of positive emotions in positive psychology. The broaden-and-build theory of positive emotions. Am Psychol. 2001;56(3):218-26. Epub 2001/04/24. PubMed PMID: 11315248.

21. Fredrickson BL, Branigan C. Positive emotions broaden the scope of attention and thought-action repertoires. Cogn Emot. 2005;19(3):313-32. Epub 2005/05/01. doi: 10.1080/02699930441000238 [doi]. PubMed PMID: 21852891; PMCID: 3156609.

22. Fredrickson BL, Cohn MA, Coffey KA, Pek J, Finkel SM. Open hearts build lives: positive emotions, induced through loving-kindness meditation, build consequential personal resources. J Pers Soc Psychol. 2008;95(5):1045-62. Epub 2008/10/29. doi: 2008-14857-004 [pii] 10.1037/a0013262 [doi]. PubMed PMID: 18954193.

23. Fredrickson BL, Joiner T. Positive emotions trigger upward spirals toward emotional well-being. Psychol Sci. 2002;13(2):172-5. Epub 2002/04/06. PubMed PMID: 11934003.

24. Fredrickson BL, Losada MF. Positive affect and the complex dynamics of human flourishing. Am Psychol. 2005;60(7):678-86. Epub 2005/10/14. doi: 2005-11834-001 [pii] 10.1037/0003-066X.60.7.678 [doi]. PubMed PMID: 16221001; PMCID: 3126111.

25. Fredrickson BL, Tugade MM, Waugh CE, Larkin GR. What good are positive emotions in crises? A prospective study of resilience and emotions following the terrorist attacks on the United States on September 11th, 2001. J Pers Soc Psychol. 2003;84(2):365-76. Epub 2003/02/15. PubMed PMID: 12585810; PMCID: 2755263.

26. L. Fredrickson B, Levenson RW. Positive Emotions Speed Recovery from the Cardiovascular Sequelae of Negative Emotions. Cognition & Emotion. 1998;12(2):191-220. doi: 10.1080/026999398379718.

27. Vaish A, Grossmann T, Woodward A. Not all emotions are created equal: the negativity bias in social-emotional development. Psychological bulletin. 2008;134(3):383.

28. Sin NL, Lyubomirsky S. Enhancing well-being and alleviating depressive symptoms with positive psychology interventions: a practice-friendly meta-analysis. J Clin Psychol. 2009;65(5):467-87. Epub 2009/03/21. doi: 10.1002/jclp.20593 [doi]. PubMed PMID: 19301241.

29. Bolier L, Haverman M, Westerhof GJ, Riper H, Smit F, Bohlmeijer E. Positive psychology interventions: a meta-analysis of randomized controlled studies. BMC public health. 2013;13(1):119.

30. Fava GA. Prevention of Recurrent Depression With Cognitive Behavioral Therapy: Preliminary Findings. Archives of General Psychiatry. 1998;55(9):816-20. doi: 10.1001/archpsyc.55.9.816.

31. Fava GA, Ruini C, Rafanelli C, Finos L, Salmaso L, Mangelli L, Sirigatti S. Well-being therapy of generalized anxiety disorder. Psychother Psychosom. 2005;74(1):26-30. doi: 10.1159/000082023. PubMed PMID: 15627853.

32. Seligman ME, Rashid T, Parks AC. Positive psychotherapy. Am Psychol. 2006;61(8):774-88.

33. Emmons RA, McCullough ME. Counting blessings versus burdens: an experimental investigation of gratitude and subjective well-being in daily life. J Pers Soc Psychol. 2003;84(2):377-89. PubMed PMID: 12585811.

34. Ducasse D, Dassa D, Courtet P, Brand‐Arpon V, Walter A, Guillaume S, Jaussent I, Olié E. Gratitude diary for the management of suicidal inpatients: A randomized controlled trial. Depression and anxiety. 2019.

35. Celano C, Beale E, Mastromauro C, Stewart J, Millstein R, Auerbach R, Bedoya C, Huffman J. Psychological interventions to reduce suicidality in high-risk patients with major depression: a randomized controlled trial. Psychological medicine. 2017;47(5):810-21.

36. A-tjak JG, Davis ML, Morina N, Powers MB, Smits JA, Emmelkamp PM. A meta-analysis of the efficacy of acceptance and commitment therapy for clinically relevant mental and physical health problems. Psychotherapy and psychosomatics. 2015;84(1):30-6.

37. Yen, Ranney ML, Tezanos KM, Chuong A, Kahler CW, Solomon JB, Spirito A. Skills to Enhance Positivity in Suicidal Adolescents: Results From an Open Development Trial. Behav Modif. 2017:145445517748559. Epub 2017/12/21. doi: 10.1177/0145445517748559. PubMed PMID: 29258328.

38. Yen S, Ranney ML, Krek M, Peters JR, Mereish E, Tezanos KM, Kahler CW, Solomon J, Beard C, Spirito A. Skills to enhance positivity in suicidal adolescents: Results from a pilot randomized clinical trial. The Journal of Positive Psychology. 2019:1-14.

39. Beck AT, Steer RA, Brown GK. Beck depression inventory-II. San Antonio. 1996;78(2):490-8.

40. Kraemer HC. Messages for Clinicians: Moderators and Mediators of Treatment Outcome in Randomized Clinical Trials. Am J Psychiatry. 2016;173(7):672-9. Epub 2016/03/19. doi: 10.1176/appi.ajp.2016.15101333. PubMed PMID: 26988629.

41. Toomey RB, Syvertsen AK, Shramko M. Transgender Adolescent Suicide Behavior. Pediatrics. 2018;142(4):e20174218. doi: 10.1542/peds.2017-4218.

42. Brent DA, Greenhill LL, Compton S, Emslie G, Wells K, Walkup JT, Vitiello B, Bukstein O, Stanley B, Posner K, Kennard BD, Cwik MF, Wagner A, Coffey B, March JS, Riddle M, Goldstein T, Curry J, Barnett S, Capasso L, Zelazny J, Hughes J, Shen S, Gugga SS, Turner JB. The Treatment of Adolescent Suicide Attempters study (TASA): predictors of suicidal events in an open treatment trial. J Am Acad Child Adolesc Psychiatry. 2009;48(10):987-96. Epub 2009/09/05. doi: 10.1097/CHI.0b013e3181b5dbe4. PubMed PMID: 19730274; PMCID: PMC2891930.

43. Esposito-Smythers C, Spirito A, Kahler CW, Hunt J, Monti P. Treatment of co-occurring substance abuse and suicidality among adolescents: a randomized trial. Journal of consulting and clinical psychology. 2011;79(6):728.

44. Wolff J, Esposito-Smythers C, Frazier E, Gomez J, Massing-Schaffer M, Nestor B, Cheek SM, Graves H, Yen S, Stout RL, Spirito A. A Randomized Pilot Effectiveness Trial of an Integrated Cognitive Behavioral Treatment Protocol for Adolescents Receiving Home-Based Services for Co-Occurring Disorders. Journal of Substance Abuse Treatment, in press

45. Spirito A, Monti PM, Barnett NP, Colby SM, Sindelar H, Rohsenow DJ, Lewander W, Myers M. A randomized clinical trial of a brief motivational intervention for alcohol-positive adolescents treated in an emergency department. The Journal of pediatrics. 2004;145(3):396-402.

46. Spirito A, Sindelar-Manning H, Colby SM, Barnett NP, Lewander W, Rohsenow DJ, Monti PM. Individual and family motivational interventions for alcohol-positive adolescents treated in an emergency department: results of a randomized clinical trial. Archives of Pediatrics & Adolescent Medicine. 2011;165(3):269-74.

47. Spirito A, Hernandez L, Marceau K, Cancilliere MK, Barnett NP, Graves HR, Rodriguez AM, Knopik VS. Effects of a brief, parent-focused intervention for substance using adolescents and their sibling. Journal of substance abuse treatment. 2017;77:156-65.

48. Greenwald AG, McGhee DE, Schwartz JL. Measuring individual differences in implicit cognition: the implicit association test. Journal of personality and social psychology. 1998;74(6):1464.

49. Quirin M, Kazén M, Kuhl J. When nonsense sounds happy or helpless: The Implicit Positive and Negative Affect Test (IPANAT). Journal of personality and social psychology. 2009;97(3):500.

50. Posner K, Oquendo MA, Gould M, Stanley B, Davies M. Columbia Classification Algorithm of Suicide Assessment (C-CASA): classification of suicidal events in the FDA's pediatric suicidal risk analysis of antidepressants. Am J Psychiatry. 2007;164(7):1035-43. Epub 2007/07/04. doi: 164/7/1035 [pii] 10.1176/appi.ajp.164.7.1035 [doi]. PubMed PMID: 17606655.

51. Kann L, Olsen EOM, McManus T, Harris WA, Shanklin SL, Flint KH, Queen B, Lowry R, Chyen D, Whittle L. Sexual Identity, Sex of Sexual Contacts, and Health-Related Behaviors among Students in Grades 9-12--United States and Selected Sites, 2015. Morbidity and Mortality Weekly Report. Surveillance Summaries. Volume 65, Number 9. Centers for Disease Control and Prevention. 2016.

52. Kaufman J, Birmaher B, Brent D, Rao U, Flynn C, Moreci P, Williamson D, Ryan N. Schedule for Affective Disorders and Schizophrenia for School-Age Children-Present and Lifetime Version (K-SADS-PL): initial reliability and validity data. J Am Acad Child Adolesc Psychiatry. 1997;36(7):980-8. Epub 1997/07/01. PubMed PMID: 9204677.

53. Burns B, Angold A, Magruder-Habib K, Costello E, Patrick M. Child and Adolescent Services Assessment. Durham, NC: Duke University; 1997.

54. Keller MB, Lavori PW, Friedman B, Nielsen E, Endicott J, McDonald-Scott P, Andreason NC. The Longitudinal Follow-up Evaluation. Archives of General Psychiatry. 1987;44:540-8.

55. Dozois DJ, Dobson KS, Ahnberg JL. A psychometric evaluation of the Beck Depression Inventory–II. Psychological assessment. 1998;10(2):83.

56. Bar-Haim Y, Lamy D, Pergamin L, Bakermans-Kranenburg MJ, van Ijzendoorn MH. Threat-related attentional bias in anxious and nonanxious individuals: A meta-analytic study. Psychological Bulletin. 2007;133(1):1-24. doi: 10.1037/0033-2909.133.1.1.

57. Brown H, Eley T, Broeren S, Macleod C, Rinck M, Hadwin J, Lester K. Psychometric properties of reaction time based experimental paradigms measuring anxiety-related information-processing biases in children. Journal of Anxiety Disorders. 2014;28(1):97-107.

58. Gibb BE, McGeary JE, Beevers CG. Attentional biases to emotional stimuli: Key components of the RDoC constructs of sustained threat and loss. American Journal of Medical Genetics Part B: Neuropsychiatric Genetics. 2016;171(1):65-80.

59. Price RB, Kuckertz JM, Siegle GJ, Ladouceur CD, Silk JS, Ryan ND, Dahl RE, Amir N. Empirical recommendations for improving the stability of the dot-probe task in clinical research. Psychological assessment. 2015;27(2):365.

60. Schmukle SC. Unreliability of the dot probe task. European Journal of Personality: Published for the European Association of Personality Psychology. 2005;19(7):595-605.

61. McCullough ME, Emmons RA, Tsang J-A. The gratitude questionnaire-six item form (GQ-6). Retrieved April. 2001;16:2010.

62. McCullough ME, Emmons RA, Tsang JA. The grateful disposition: a conceptual and empirical topography. J Pers Soc Psychol. 2002;82(1):112-27. Epub 2002/01/29. PubMed PMID: 11811629.

63. Diener E, Emmons RA, Larsen RJ, Griffin S. The Satisfaction With Life Scale. J Pers Assess. 1985;49(1):71-5. Epub 1985/02/01. doi: 10.1207/s15327752jpa4901_13. PubMed PMID: 16367493.

64. Proctor E, Silmere H, Raghavan R, Hovmand P, Aarons G, Bunger A, Griffey R, Hensley M. Outcomes for implementation research: conceptual distinctions, measurement challenges, and research agenda. Administration and Policy in Mental Health and Mental Health Services Research. 2011;38(2):65-76.

65. Weiner BJ, Lewis CC, Stanick C, Powell BJ, Dorsey CN, Clary AS, Boynton MH, Halko H. Psychometric assessment of three newly developed implementation outcome measures. Implementation Science. 2017;12(1):108.

66. Charmaz K. Constructing grounded theory: sage; 2014.

67. Solutions Q. NUD-IST Vivo (Nvivo)[Computer software]1999.

68. Fetters MD, Curry LA, Creswell JW. Achieving integration in mixed methods designs-principles and practices. Health Serv Res. 2013;48(6 Pt 2):2134-56. Epub 2013/11/28. doi: 10.1111/1475-6773.12117. PubMed PMID: 24279835; PMCID: PMC4097839.

69. Hsieh H-F, Shannon SE. Three approaches to qualitative content analysis. Qualitative health research. 2005;15(9):1277-88.

70. Curran GM, Bauer M, Mittman B, Pyne JM, Stetler C. Effectiveness-implementation hybrid designs: combining elements of clinical effectiveness and implementation research to enhance public health impact. Med Care. 2012;50(3):217-26. Epub 2012/02/09. doi: 10.1097/MLR.0b013e3182408812. PubMed PMID: 22310560; PMCID: PMC3731143.

71. Powell BJ, Waltz TJ, Chinman MJ, Damschroder LJ, Smith JL, Matthieu MM, Proctor EK, Kirchner JE. A refined compilation of implementation strategies: results from the Expert Recommendations for Implementing Change (ERIC) project. Implementation Science. 2015;10(1):21.

72. Proctor EK, Powell BJ, McMillen JC. Implementation strategies: recommendations for specifying and reporting. Implementation Science. 2013;8(1):139.

73. Elwy AR, Wasan AD, Gilman AG, Johnston KL, Dodds N, McFarland C, Greco CM. Using Formative Evaluation Methods to Improve Clinical Implementation Efforts: Description and an Example. Psychiatry Research. 2019:112532.

74. Stirman SW, Miller CJ, Toder K, Calloway A. Development of a framework and coding system for modifications and adaptations of evidence-based interventions. Implementation Science. 2013;8(1):65.

75. Schulz KF, Altman DG, Moher D. CONSORT 2010 Statement: updated guidelines for reporting parallel group randomised trials. BMJ. 2010;340.

76. Ware JH, Harrington D, Hunter DJ, D'Agostino RB. Missing data. N Engl J Med. 2012;367:1353-4.

77. Baron RM, Kenny DA. The moderator-mediator variable distinction in social psychological research: conceptual, strategic, and statistical considerations. J Pers Soc Psychol. 1986;51(6):1173-82. Epub 1986/12/01. PubMed PMID: 3806354.

78. Jo B. Causal inference in randomized experiments with mediational processes. Psychological methods. 2008;13(4):314.

79. Maldonado G, Greenland S. Estimating causal effects. International journal of epidemiology. 2002;31(2):422-9. Epub 2002/05/01. PubMed PMID: 11980807.

80. Shrout PE, Bolger N. Mediation in experimental and nonexperimental studies: new procedures and recommendations. Psychological methods. 2002;7(4):422.

81. Stanley B, Brown GK. Safety Planning Intervention: A Brief Intervention to Mitigate Suicide Risk. Cognitive and Behavioral Practice. 2012;19(2):256-64. doi: <https://doi.org/10.1016/j.cbpra.2011.01.001>.

82. Brent DA, Poling KD, Goldstein TR. Treating Depressed and Suicidal Adolescents: A Clinician's Guide: Guilford Publications; 2011.

83. Wolff JC, Frazier EA, Weatherall SL, Thompson AD, Liu RT, Hunt JI. Piloting of COPES: An Empirically Informed Psychosocial Intervention on an Adolescent Psychiatric Inpatient Unit. Journal of Child and Adolescent Psychopharmacology. 2018;28(6):409-14. doi: 10.1089/cap.2017.0135.
